# Pretargeted PET Imaging with a TCO-Conjugated Anti-CD44v6 Chimeric mAb U36 and [^89^Zr]Zr-DFO-PEG_5_-Tz

**DOI:** 10.1021/acs.bioconjchem.2c00164

**Published:** 2022-04-20

**Authors:** Dave Lumen, Danielle Vugts, Marion Chomet, Surachet Imlimthan, Mirkka Sarparanta, Ricardo Vos, Maxime Schreurs, Mariska Verlaan, Pauline A. Lang, Eero Hippeläinen, Wissam Beaino, Albert D. Windhorst, Anu J. Airaksinen

**Affiliations:** † Department of Chemistry, Radiochemistry, University of Helsinki, FI-00014 Helsinki, Finland; ‡ Amsterdam UMC, Vrije Universiteit Amsterdam, Radiology & Nuclear Medicine, Cancer Center Amsterdam, De Boelelaan 1117, 1081 HV Amsterdam, The Netherlands; § HUS Medical Imaging Center, Clinical Physiology and Nuclear Medicine, University of Helsinki and Helsinki University Hospital, 00029 HUS Helsinki, Finland; ∥ Turku PET Centre, Department of Chemistry, University of Turku, 20520 Turku, Finland

## Abstract

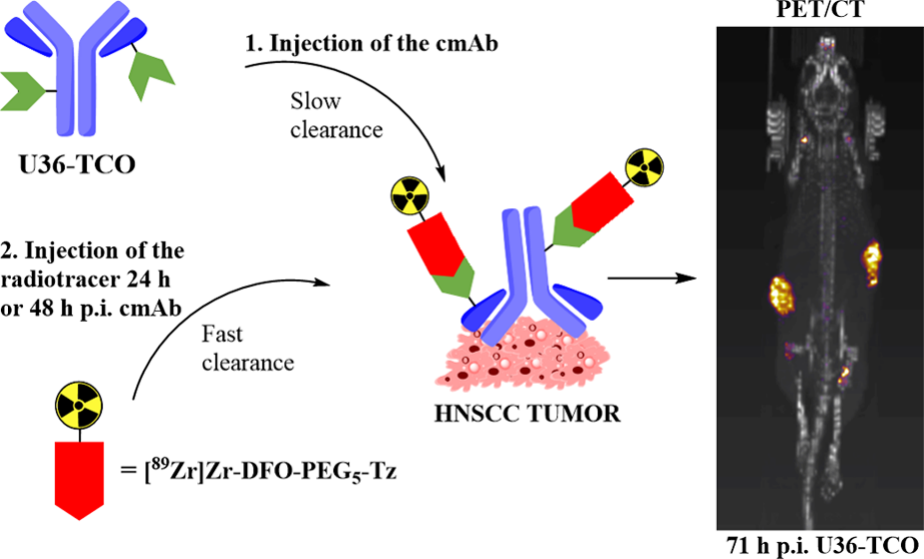

The recent advances in the production of engineered antibodies have facilitated the development and application of tailored, target-specific antibodies. Positron emission tomography (PET) of these antibody-based drug candidates can help to better understand their *in vivo* behavior. In this study, we report an *in vivo* proof-of-concept pretargeted immuno-PET study where we compare a pretargeting vs targeted approach using a new ^89^Zr-labeled tetrazine as a bio-orthogonal ligand in an inverse electron demand Diels–Alder (IEDDA) *in vivo* click reaction. A CD44v6-selective chimeric monoclonal U36 was selected as the targeting antibody because it has potential in immuno-PET imaging of head-and-neck squamous cell carcinoma (HNSCC). Zirconium-89 (*t*_1/2_ = 78.41 h) was selected as the radionuclide of choice to be able to make a head-to-head comparison of the pretargeted and targeted approaches. [^89^Zr]Zr-DFO-PEG_5_-Tz ([^89^Zr]Zr-**3**) was synthesized and used in pretargeted PET imaging of HNSCC xenografts (VU-SCC-OE) at 24 and 48 h after administration of a *trans*-cyclooctene (TCO)-functionalized U36. The pretargeted approach resulted in lower absolute tumor uptake than the targeted approach (1.5 ± 0.2 vs 17.1 ± 3.0% ID/g at 72 h p.i. U36) but with comparable tumor-to-non-target tissue ratios and significantly lower absorbed doses. In conclusion, anti-CD44v6 monoclonal antibody U36 was successfully used for ^89^Zr-immuno-PET imaging of HNSCC xenograft tumors using both a targeted and pretargeted approach. The results not only support the utility of the pretargeted approach in immuno-PET imaging but also demonstrate the challenges in achieving optimal *in vivo* IEDDA reaction efficiencies in relation to antibody pharmacokinetics.

## Introduction

**Figure 1:**
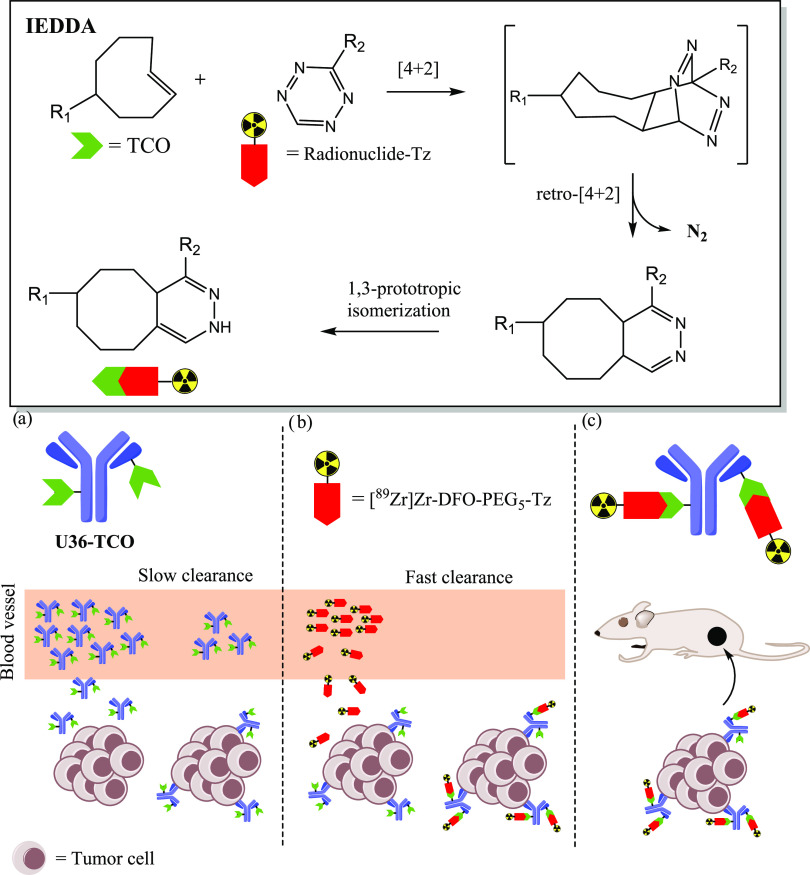
Pretargeting method based on an inverse electron demand Diels–Alder (IEDDA) ligation between *trans*-cyclooctene (TCO) and tetrazine. In the first step (a), a TCO-conjugated antibody is administered and allowed to reach the target, while unbound antibodies are slowly cleared from the circulation. In the second step (b), a radiolabeled tetrazine is administered and it reacts with the TCO-antibody. Unreacted tetrazine molecules are cleared fast from circulation. The radiolabeled antibody (c) is now visible compared to the nontarget tissue since most of the detected radioactivity signals originate from the tumor.

Quantitative positron emission tomography (PET) imaging can be used in preclinical as well as clinical research and provides important information about the pharmacokinetics of monoclonal antibodies (mAbs) and derivatives thereof, particularly with respect to the kinetics of tumor accumulation and washout from nontarget tissues.^1^ During the last decades, many antibodies have been developed for cancer diagnosis and treatment, and recent advances in the production of tailored antibodies for specific targets have provided several new radioimmunoconjugate candidates for immuno-PET imaging.^2−4^ These second-generation radioimmunoconjugates can be grouped into different categories: (i) antibody–drug conjugates (ADCs), designed to release a drug when reaching its target;^5,6^ (ii) multispecific mAbs, recognizing two or more targets;^7^ (iii) glycoengineered mAbs, which are modified to enhance the antibody-dependent cytotoxicity;^8^ and (iv) mAb fragments and nanobodies to tailor the radioimmunoconjugate pharmacokinetics.^9^ The relatively slow pharmacokinetics of antibodies require that the radioactive half-life of the isotope must be compatible with the biological half-life of the mAb. In practice, this means that for immuno-PET imaging the antibodies are often labeled with isotopes with long, even multiday physical half-lives such as ^89^Zr (78.41 h), ^64^Cu (12.70 h), and ^124^I (4.18 d),^10−12^ which allows for the detection of the radiolabeled antibodies after accumulation at the tumor and clearance from the circulation.^13^ It usually takes several days until nonbound antibodies are cleared from the circulation, and the optimal target-to-non-target (T:NT) values are obtained for imaging.^14,15^ The administered radioactive dose can therefore be high. The levels of radiolabeled mAbs in blood can be reduced using special clearing agents;^16^ however, this does not solve the problem of slow accumulation kinetics of mAbs in the tumor. Achieving high target-to-non-target values more rapidly would minimize the lag time needed between the radiotracer injection and the PET imaging, reducing exposure of the patient to radioactivity and the effective dose. Significant efforts have been dedicated to overcome these obstacles through the development of engineered antibody variants with faster pharmacokinetics and pretargeted approaches for radiolabeling the antibodies *in vivo* after their administration and peak accumulation to the target site.^12^ Recently, *in vivo* click reactions based on the bio-orthogonal inverse electron demand Diels–Alder ligation (IEDDA) between dienophile-functionalized antibodies and small-molecule radioligands based on tetrazine structures have obtained high interest.^17−22^ Pretargeted immuno-PET imaging would bring significant advantages: reducing the radioactive exposure of the patients and allowing the use of the short half-live radionuclides for imaging purposes (Figure 1).^12,23^ The preclinical proof of concept of the two-step pretargeted immuno-PET imaging and radioimmunotherapy with IEDDA have been successfully achieved by several research groups.^17,24−27^

Bio-orthogonal click reactions are specific and selective reactions that can take place under physiological conditions and rapidly react even at low concentrations *in vivo*. Fast reaction kinetics and selectivity have made them a favorable choice for effective *in vivo* radiolabeling methods for pretargeted imaging and therapy.^28^ The IEDDA ligation between olefins or alkynes (e.g., *trans*-cyclooctene or TCO) and 1,2,4,5-tetrazines (e.g., tetrazine or Tz) is a selective, fast, high-yielding, biocompatible, and bio-orthogonal reaction, in which the reaction counterparts will undergo two concerted reactions to afford a coupling product under the formation of a pyridazine and dinitrogen (Figure 1). Reaction between TCO and Tz holds one of the fastest reaction kinetics from all click chemistry methods, which makes them ideal functional groups for *in vivo* applications. Rate constants for the reaction between tetrazine and TCO can exceed 100,000 M^–1^ s^–1^, orders of magnitude faster than either the Staudinger or strain-promoted azide–alkyne cycloaddition ligations.^29^ Rossin et al. used the IEDDA for the first time for pretargeted SPECT imaging, and the first pretargeted PET study was reported by Weissleder and Lewis.^18,30^ TCO isomerizes quickly to a less reactive *cis*-cyclooctene (CCO) *in vivo* unless conjugated to a macromolecule; therefore, most of the published pretargeting studies are based on the IEDDA ligation between a TCO-conjugated antibody and a small-molecular tetrazine carrying the radiolabel.

In this study, a ^89^Zr-labeled tetrazine ([^89^Zr]Zr-DFO-PEG_5_-Tz, [^89^Zr]Zr-**3**) was developed and utilized as a tool for investigation and comparison of targeted and pretargeted PET imaging of head-and-neck squamous cell carcinoma (VU-SCC-OE) xenografts using an anti-CD44v6 chimeric mAb (cmAb) U36.^31^ U36 was chosen for the study because it has shown high and selective tumor uptake in head-and-neck squamous cell carcinoma (HNSCC) patients and it internalizes into cells only to a limited extent.^31^ The splice variant v6 of the cell membrane glycoprotein CD44 (CD44v6) is expressed only in a few normal epithelial tissues (e.g., thyroid and prostate gland),^32^ but it plays a significant role in solid tumor growth and metastasis development. For the HNSCC, >96% of tumors show CD44v6 expression by at least 50% of the cells.^33^ In addition to squamous cell carcinomas, CD44v6 is overexpressed in adenocarcinomas and ovarian cancer and in hematological tumors.^34−36^ Expression of CD44v6 in tumors has been imaged by several research groups using U36 or its variants after radiolabeling it with different long-living radionuclides.^37−40^ In this study, U36 was conjugated with *trans*-cyclooctene and the conjugation ratio was optimized with biodistribution studies. TCO–U36 was radiolabeled *in vitro* and *in vivo* using [^89^Zr]Zr-**3**, and the uptake levels in VU-SCC-OE tumors were quantified with PET-CT/MRI and *ex vivo* biodistribution studies.

## Results

### Synthesis of [^89^Zr]Zr-DFO-PEG_5_-Tz ([^89^Zr]Zr-3)

**Scheme 1:**
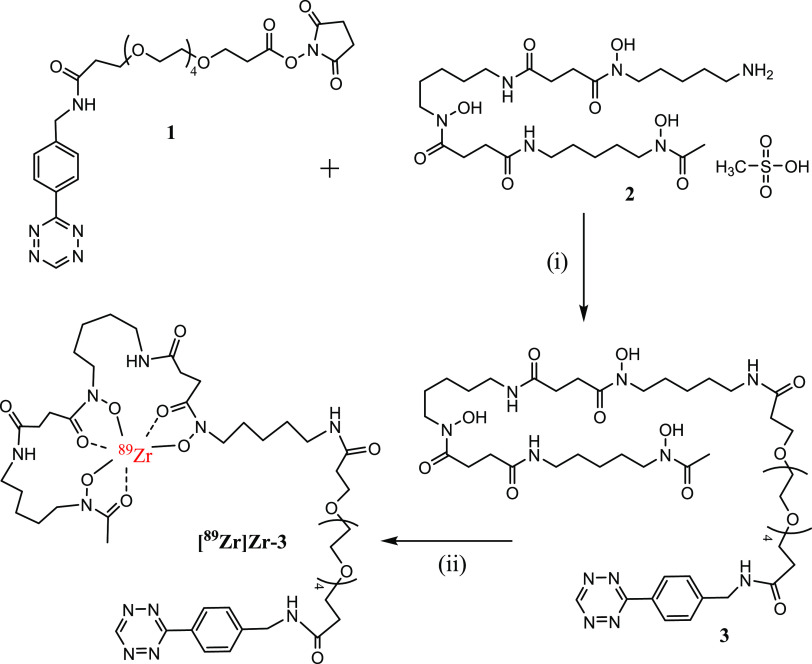
Schematic Representation of the Chemical Synthesis of **3** and Radiosynthesis of [^89^Zr]Zr-DFO-PEG_5_-Tz ([^89^Zr]Zr-**3**) Reaction conditions: (i) dimethyl formamide (DMF), Et_3_N, hexafluorophosphate (HATU), overnight reaction at room temperature (rt) in dark conditions, (ii) ^89^Zr-oxalate, Na_2_CO_3_, oxalic acid, 4-(2-hydroxyethyl)-1-piperazineethanesulfonic acid (HEPES) buffer (pH 7) at room temperature.

DFO-PEG_5_-Tz (**3**) was synthesized from tetrazine-PEG_5_-NHS ester (**1**) and DFO mesylate (**2**) under mild reaction conditions followed by a C18 SepPak purification, yielding **3** as a pink solid with a 31 ± 11% yield (*n* = 3) (Scheme 1). The purification step had a great effect on the yield since the product tended to attach to the SPE matrix. Compound **3** was radiolabeled with [^89^Zr]Zr-oxalate, yielding [^89^Zr]Zr-DFO-PEG_5_-Tz ([^89^Zr]Zr-**3**) with good radiochemical yields (RCYs = 80 ± 16%, *n* = 6) when 0.94–13.2 nmol (1–14 μg) of the chelator (**3**) was used. Radiochemical stability of [^89^Zr]Zr-**3** was assessed with iTLC and high-performance liquid chromatography (HPLC) in the formulation buffer (10% EtOH in saline + 0.1% Tween + 20 mM gentisic acid, pH 5.2) at 4, 24, and 48 h (Figure S7). Stability of [^89^Zr]Zr-**3** was excellent with >98% intact radiotracer in the formulation buffer at 4 h and >96% at 48 h (*n* = 2).

### *In Vitro* Radiolabeling of TCO–U36

**Scheme 2:**
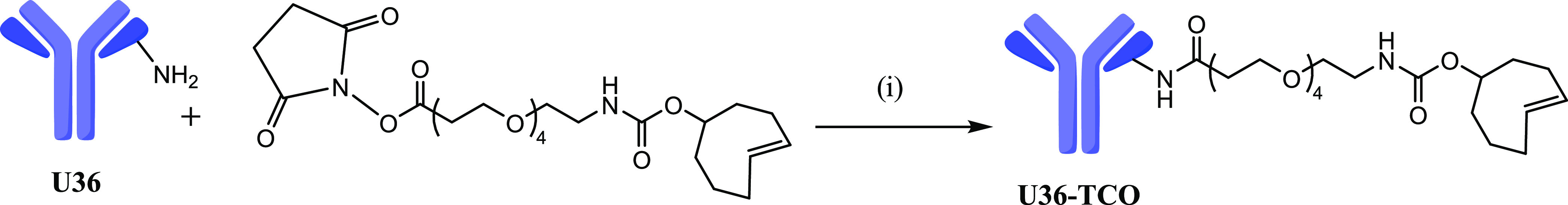
Synthetic Scheme of TCO-Functionalized U36 Antibody (TCO–U36) Reaction conditions: (i) PBS (pH 8.5), room temperature, overnight.

U36 was conjugated with TCO-PEG_4_-NHS (**5**, 10–40 equiv) at room temperature (rt) overnight, followed by subsequent purification with a PD-10 desalting column (Scheme 2) using phosphate-buffered saline (PBS) as an eluent. The obtained TCO-to-U36 ratios were determined after isolation using a matrix-assisted laser desorption ionization time-of-flight mass spectrometry (MALDI-TOF-MS) confirming TCO-to-U36 ratios between 6.2 and 27.2 depending on the excess of **5** added in the reaction. The isolated TCO–U36 was radiolabeled with [^89^Zr]Zr-**3** in a buffer solution at rt using a [^89^Zr]Zr-**3**-to-U36 ratio of 1:1. Unbound [^89^Zr]Zr-**3** was removed with a PD-10 column yielding [^89^Zr]Zr-**3**–TCO–U36 with a high RCY of 85 ± 4% and RCP > 99%. The yield was not dependent on the TCO-to-U36 ratio, which varied between 6.2 and 27.2. However, if less than 0.5 mg of U36 was used, losses during the purification and concentration increased, lowering the RCY closer to 70%.

### Immunoreactivity of [^89^Zr]Zr-3–TCO–U36 with CD44v6

Immunoreactivity of [^89^Zr]Zr-**3**–TCO–U36 was determined using CD44v6-coated beads using TCO-conjugated U36 with the highest TCO-to-U36 ratio (27:1). Despite the high TCO-to-U36 ratio, immunoreactivity was well preserved with a 91.6 ± 1.3% immunoreactivity corrected for nonspecific binding at a CD44v6 bead concentration of 1.6 × 10^6^/mL (*n* = 3) (Figure S1).

### *Ex Vivo* Biodistribution of [^89^Zr]Zr-3

**Figure 2:**
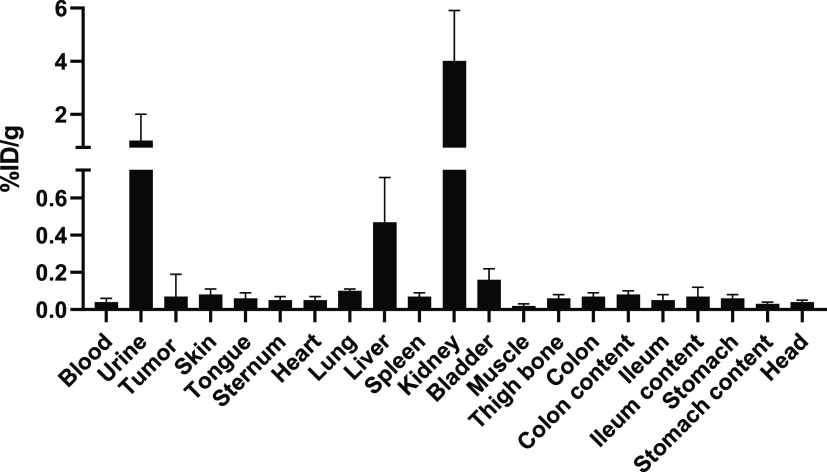
*Ex vivo* biodistribution of [^89^Zr]Zr-**3** (350 ± 50 kBq i.v., in 100 μL of 10% EtOH in saline + 0.1% Tween + 20 mM gentisic acid, pH 5.2) at 24 h p.i. in VU-SCC-OE tumor-bearing mice (*n* = 4). The results demonstrate fast clearance *via* the urinary system and low nonspecific tracer accumulation in healthy organs and in the tumor. The results are presented as % ID/g (mean ± standard deviation, SD).

Pharmacokinetics of the radiolabeled tetrazine [^89^Zr]Zr-**3** was determined in athymic nude NMRI mice (*n* = 3 per time point) at 1, 4, and 24 h after i.v. administration of the tracer (350 ± 50 kBq, 0.7 μg, 0.66 nmol in 100 μL of 10% EtOH in saline + 0.1% Tween + 20 mM gentisic acid, pH 5.2) (Figure S2). The level of nonspecific accumulation of [^89^Zr]Zr-**3** into tumor was determined in VU-SCC-OE tumor-bearing mice (*n* = 4) at 24 h after i.v. administration of the tracer. [^89^Zr]Zr-**3** exhibited fast clearance and elimination mainly *via* kidneys to urine, and less than 0.5% ID/g residual radioactivity was observed in other organs and in the tumor at 24 h p.i. (Figure 2).

### Biological Evaluation of [^89^Zr]Zr-3 for Labeling of TCO–U36 in VU-SCC-OE Xenografts with a TCO-to-U36 Ratio of 27:1

**Figure 3:**
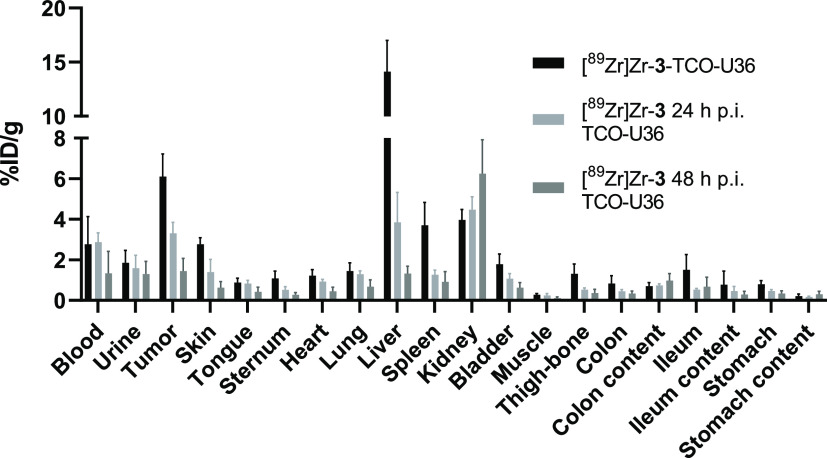
*Ex vivo* biodistribution of *in vitro* and *in vivo* [^89^Zr]Zr-**3**-labeled TCO–U36 (0.1 mg, 0.66 nmol) at 72 h p.i. cmAb with a TCO-to-U36 ratio of 27:1 in VU-SCC-OE tumor-bearing mice. For the *in vivo* pretargeting, [^89^Zr]Zr-**3** was injected 24 and 48 h p.i. of TCO–U36 (4.1 ± 0.3 and 3.9 ± 0.5 MBq, 0.7 μg, 0.66 nmol, respectively) ([^89^Zr]Zr-**3**-to-U36 ratio 1:1). The results are presented as % ID/g (mean ± SD, *n* = 4).

*In vivo* IEDDA reactivity of [^89^Zr]Zr-**3** was tested first in VU-SCC-OE xenografts by the pretargeted approach and TCO-conjugated U36 antibody with the highest 27:1 TCO-to-U36 ratio. Mice injected with *in vitro*-radiolabeled [^89^Zr]Zr-**3**–TCO–U36 were used as a control group. The results revealed that the pharmacokinetics of the antibody were significantly altered due to the excessive TCO conjugation (Figure 3 and Table S2). Liver uptake for the *in vitro*-labeled [^89^Zr]Zr-**3**–TCO–U36 was high (14.1 ± 2.9% ID/g at 72 h p.i.), and tumor uptake was lower (6.1 ± 1.1% ID/g at 72 h p.i.) compared to the results previously reported by Vugts et al. using the same mAb dose (0.1 mg, azide conjugation ratio 4:1; liver: 3.9 ± 0.4% ID/g and tumor: 23.1 ± 3.4% ID/g at 72 h p.i.).^41^ However, the initial results confirmed successful *in vivo* IEDDA reaction with the highest tumor uptake of 3.3 ± 0.5% ID/g at 72 h when the tracer [^89^Zr]Zr-**3** was injected at 24 h p.i. TCO–U36 and 1.5 ± 0.6% ID/g when injected at 48 h p.i. TCO–U36. The results indicate that the maximum 50% of TCO–U36 reaching the tumor at 72 h was radiolabeled *in vivo* since tumor accumulation of the *in vitro*-labeled [^89^Zr]Zr-**3**–TCO–U36 was 6.11 ± 1.12% ID/g at 72 h. It was therefore evident that further optimization of the TCO-to-mAb ratio was needed for minimizing the effect of the TCO conjugation on the pharmacokinetics of the antibody.

### *Ex Vivo* Biodistribution of [^89^Zr]Zr-3–TCO–U36 with Different TCO Conjugation Ratios in Non-Tumor-Bearing Animals

**Figure 4:**
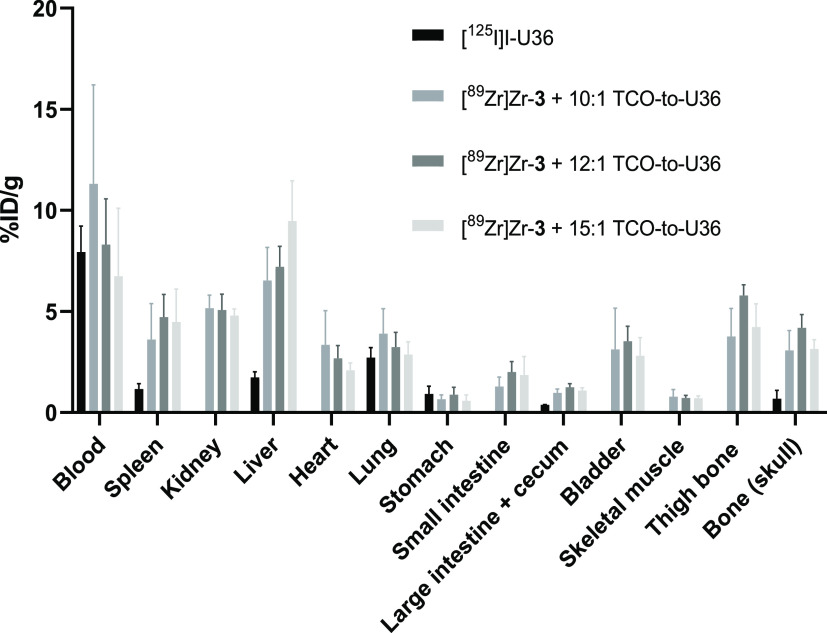
*Ex vivo* biodistribution of [^125^I]I-U36 (350 ± 50 kBq, 0.1 mg, 0.66 nmol) and *in vitro*-radiolabeled [^89^Zr]Zr-**3**–TCO–U36 (150 ± 50 kBq, 0.1 mg, 0.66 nmol) with different TCO-to-U36 ratios 72 h after injection to athymic nude NMRI mice. The results are presented as % ID/g (mean ± SD; *n* = 4).

Biodistribution of the [^89^Zr]Zr-**3**-labeled U36 was investigated with varying TCO-to-U36 ratios and compared to the biodistribution of ^125^I-labeled U36 without any TCO groups attached. *Ex vivo* biodistribution at 72 h p.i. showed clearly how the TCO-to-U36 ratio affected the liver uptake of the antibody and how the blood radioactivity levels increased with decreasing antibody accumulation in the liver (Figure 4). With a TCO-to-U36 ratio of 10:1, the lowest liver uptake and the highest radioactivity in the circulation were obtained.

**Figure 5:**
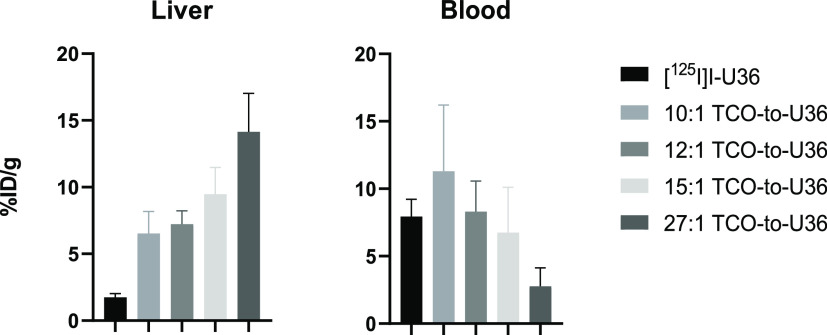
Comparison of radioactivity (% ID/g) in liver and blood for ^125^I-labeled U36 and *in vitro*-radiolabeled [^89^Zr]Zr-**3**–TCO–U36 with different TCO-to-U36 ratios at 72 h p.i. in athymic nude NMRI mice and in mice bearing VU-SCC-OE xenografts (27:1 TCO-to-U36) (columns denote mean ± SD, *n* = 4).

A clear correlation was observed between the increased liver uptake and decreased blood concentrations when more TCO moieties were conjugated to U36 (Pearson correlation coefficient R for liver = 99.3 and for blood = −68.6) (Figure 5). The effect of small-molecule conjugation on the U36 antibody pharmacokinetics was surprisingly high compared to the finding of the reported study by Vugts et al. with a phenolic PEG_5_-triazide-conjugated U36, where the influence of the azide conjugation to liver accumulation and to clearance from blood was less prominent even with a ratio of 15 azides on 1 U36.^41^ Therefore, we decided to repeat the pretargeted PET study with even a lower TCO-to-U36 ratio than 10:1 with the goal of further decreasing the observed liver uptake.

### *In Vivo* Evaluation of TCO–U36 with a 6:1 TCO-to-U36 Ratio in VU-SCC-OE Xenografts

**Figure 6:**
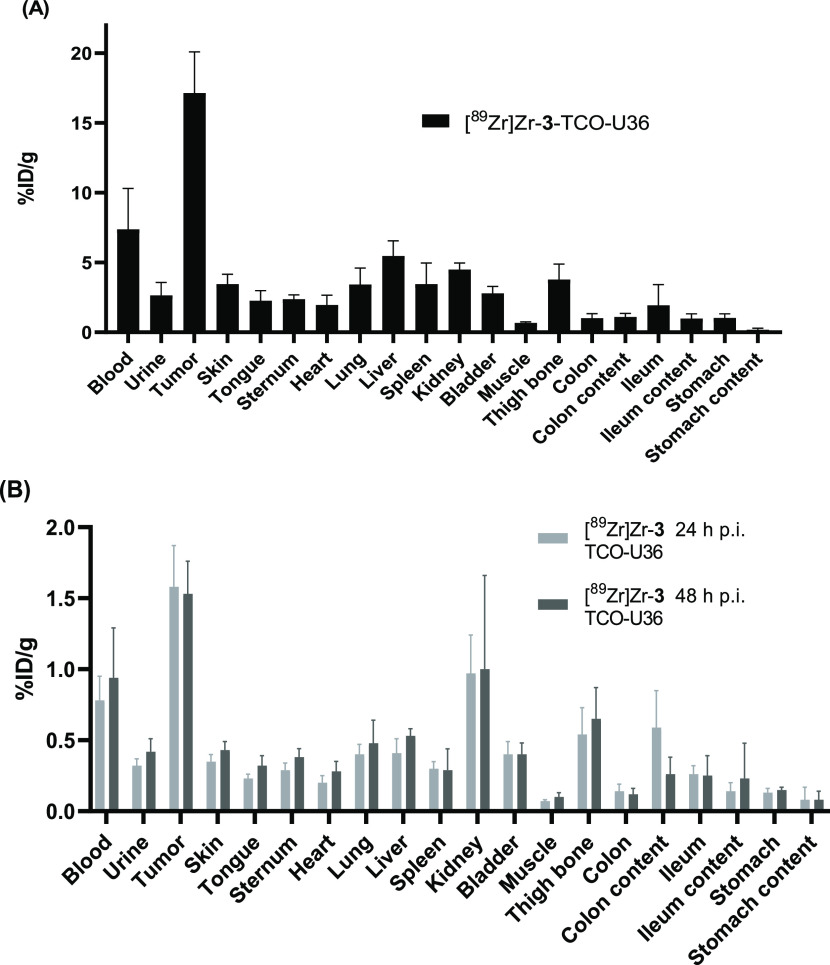
*Ex vivo* biodistribution of (A) *in vitro*-labeled [^89^Zr]Zr-**3**–TCO–U36 (3.0 ± 0.3 MBq, 0.1 mg, 0.66 nmol) and (B) *in vivo* ([^89^Zr]Zr-**3**) (2.5 ± 0.2 MBq, 0.7 μg, 0.66 nmol)-labeled U36 (0.1 mg, 0.66 nmol, 6:1 TCO-to-U36) at 72 h p.i. of cmAb in VU-SCC-OE xenografts ([^89^Zr]Zr-**3**-to-U36 ratio 1:1). The results are presented as % ID/g (mean ± SD, *n* = 4).

Using the same experimental setup as used in the initial biological evaluation, the *ex vivo* biodistribution data showed improved pharmacokinetics of [^89^Zr]Zr-**3**–TCO–U36 with a typical, high tumor accumulation of 17.1 ± 3.0% ID/g and a low liver uptake of 5.5 ± 1.1% ID/g at 72 h p.i. (Figure 6A and Table S2). However, tumor uptake in the pretargeted approach was lower: 1.6 ± 0.3% ID/g when [^89^Zr]Zr-**3** was injected at 24 h p.i. of U36 and 1.5 ± 0.2% ID/g when injected at 48 h p.i. of U36 (Figure 6B). The observed decrease in the tumor uptake was statistically significant when compared to the results obtained with the high TCO-to-U36 ratio (27:1) construct, 3.3 ± 0.5% ID/g at 72 h. Obviously, reducing the number of TCO groups conjugated to U36 had a significant influence on the *in vivo* radiolabeling efficiency of the tumor antigen-bound TCO–U36, which dropped below 10% (1.6 ± 0.3% ID/g in tumor at 72 h vs 17.1 ± 3.0% ID/g in tumor with *in vitro*-labeled [^89^Zr]Zr-**3**–TCO–U36).

Although the tumor uptake values were significantly lower with the pretargeted approach, the same tumor-to-background ratios were achieved when compared to the *in vitro*-labeled U36 (Table 1). For the *in vitro*-labeled U36, the tumor-to-muscle ratio was 25.67 ± 6.30, and for the *in vivo* pretargeting, the ratio was 23.49 ± 6.22 when the tracer was injected 24 h p.i. of the TCO–U36. The tumor uptake was slightly lower when the tracer was injected 48 h p.i. of TCO–U36, resulting in a lower tumor-to-muscle ratio of 15.56 ± 6.57.

**Table 1:** *Ex Vivo* Biodistribution at 72 h p.i. of cmAb in VU-SCC-OE Tumor, Muscle, Liver, and Blood (% ID/g) and Calculated Tumor-to-Muscle (T/M), Tumor-to-Liver (T/L), and Tumor-to-Blood (T/B) Ratios for *in Vivo*- and *in Vitro*-Labeled U36 Antibodies (6:1 TCO-to-U36)^a^

	[^89^Zr]Zr-3 injection 24 h p.i. TCO–U36	[^89^Zr]Zr-3 injection 48 h p.i. TCO–U36	*in vitro*-labeled [^89^Zr]Zr-3–TCO–U36
tumor	1.58 ± 0.29	1.53 ± 0.23	17.14 ± 2.95
muscle	0.07 ± 0.01	0.10 ± 0.03	0.67 ± 0.08
liver	0.41 ± 0.10	0.53 ± 0.05	5.47 ± 0.08
blood	0.78 ± 0.17	0.94 ± 0.35	7.37 ± 2.93
T/M ratio	23.49 ± 6.22	15.56 ± 6.57	25.67 ± 6.30
T/L ratio	3.82 ± 1.46	2.88 ± 0.60	3.13 ± 0.63
T/B ratio	2.03 ± 0.71	1.63 ± 0.88	2.33 ± 1.40

a Data is given as mean ± standard deviation.

**Figure 7:**
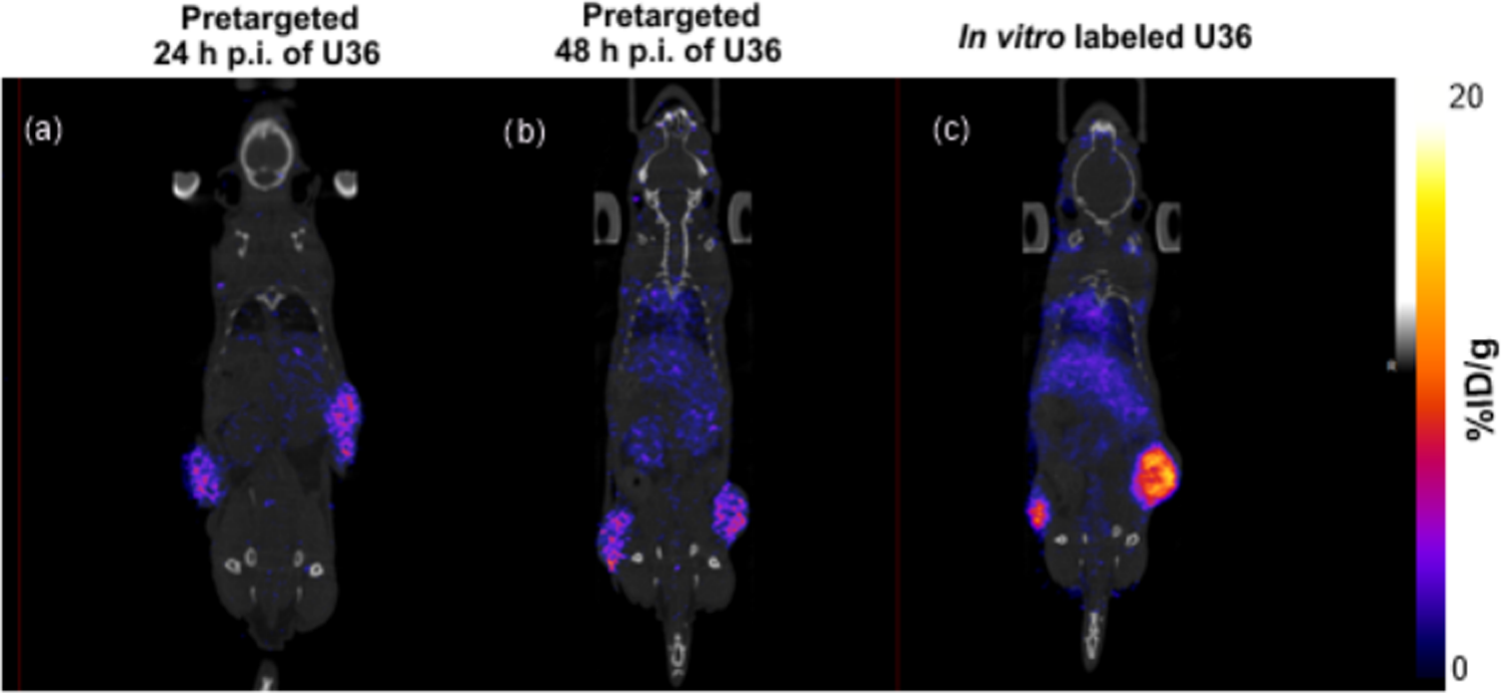
Coronal PET/CT images for all groups at 71 h p.i. of the U36 antibody administration in VU-SCC-OE xenografts; [^89^Zr]Zr-**3** was injected (a) 24 h or (b) 48 h p.i. of TCO–U36 ([^89^Zr]Zr-**3**-to-U36 ratio 1:1). The third group (c) was injected with *in vitro-*labeled [^89^Zr]Zr-**3**–TCO–U36 at *t* = 0.

**Figure 8:**
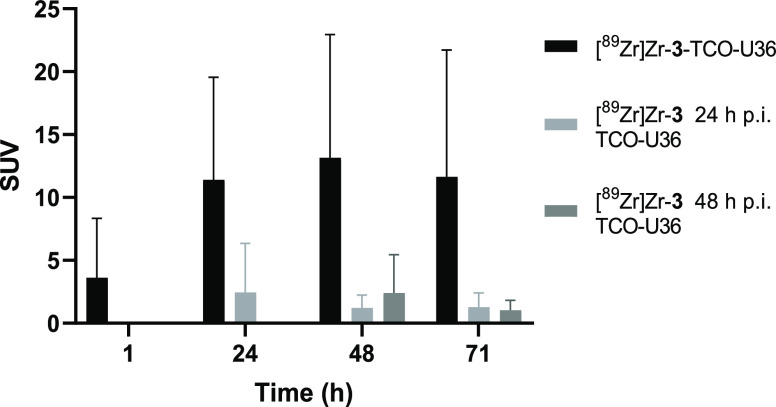
Standardized uptake values (SUVs) in the VU-SCC-OE xenograft tumors for all groups at 1, 24, 48, and 71 h after the U36 injection. The results are presented as SUV (mean ± SD, *n* = 4).

Despite the lower activity concentration in the pretargeted tumors, the tumors were clearly visible by PET-computed tomography (PET/CT) due to the low background activity (Figure 7). Tumor activities were quantified by delineating region of interests around the tumors and by calculating standardized uptake values (SUVs) for all groups at 1, 24, 48, and 71 h after the U36 injection (Figure 8). The tumor volumes varied from 31 to 793 mm^3^, and the heterogeneous structure of the tumors caused some additional challenge for the image analysis and calculation of the SUVs. Due to the structural heterogeneity (necrotic core poorly perfused), the activity concentrations varied significantly between the tumors, resulting in high variation of the SUVs between tumors from the same group. In general, small tumors (<100 mm^3^) had clearly higher activity concentration compared to the larger ones (Table S1).

Volume-of-interest (VOI) values from the PET/CT images were used to estimate absorbed doses in selected organs. The dosimetry calculations revealed significantly lower absorbed doses for the pretargeted groups ([^89^Zr]Zr-**3** injection 24 or 48 h p.i. TCO–U36) compared to those for the *in vitro*-labeled U36 ([^89^Zr]Zr-**3**–TCO–U36) (Table 2). Especially, for the few important organs, the absorbed dose difference was significant between the pretargeted U36 and the *in vitro*-labeled U36 groups, for example, in the heart (0.086 and 0.072 vs 0.471 for 24 h pretargeted, 48 h pretargeted, and *in vitro*-labeled groups, respectively), liver (0.123 and 0.082 vs 0.970), and spleen (0.057 and 0.054 vs 0.395). There was also a considerable difference between the two approaches when considering the absorbed dose to the bone. Dose values for red marrow and osteogenic cells were approximately 5 times lower with the pretargeted approach. The dose estimations for the *in vitro-*labeled U36 were in line with the results that were reported by Börjesson and co-workers with ^89^Zr-labeled U36 in humans.^42^ Although the values from the human study were higher (liver 1.25 vs 0.97, kidneys 0.82 vs 0.35, spleen 0.67 vs 0.40 and total body 0.44 vs 0.19), it can be explained partly due to their longer experimental setup (133 h).

**Table 2:** Dosimetry Calculation for Pretargeted Groups ([^89^Zr]Zr-**3** Injection 24 or 48 h p.i. TCO–U36) and the *In Vitro*-Labeled U36 (6:1 TCO-to-U36)^a^

target organ	[^89^Zr]Zr-3 injection 24 h p.i. TCO–U36	[^89^Zr]Zr-3 injection 48 h p.i. TCO–U36	*in vitro*-labeled [^89^Zr]Zr-3–TCO–U36
large intestine	0.047	0.046	0.270
small intestine	0.047	0.088	0.493
stomach wall	0.050	0.039	0.273
heart	0.086	0.077	0.471
kidneys	0.110	0.071	0.345
liver	0.123	0.082	0.970
lungs	0.036	0.028	0.209
pancreas	0.056	0.047	0.336
red marrow	0.043	0.041	0.203
osteogenic cells	0.056	0.045	0.314
spleen	0.057	0.054	0.395
bladder	0.059	0.067	0.195
total body	0.039	0.037	0.188
effective dose	0.042	0.038	0.223

a Mean organ-absorbed doses and total body effective dose are expressed in mGy/MBq and mSv/MBq, respectively.

## Discussion

In this study, we investigated the pretargeted PET imaging of VU-SCC-OE xenografts utilizing the IEDDA reaction between a zirconium-89-labeled tetrazine ([^89^Zr]Zr-**3**) and a TCO-functionalized anti-CD44v6 antibody U36. The relatively long half-life (*t*_1/2_ = 78.41 h) of zirconium-89 enabled the direct comparison of the tumor targeting *in vivo* with *in vitro*-labeled U36 and after pretargeting of TCO-modified U36. U36 was chosen for the study because it has shown high and selective tumor uptake in head-and-neck squamous cell carcinoma patients and it internalizes into cells only to a limited extent.^31^ Both properties are favorable for successful pretargeting. *In vitro* radiolabeling between [^89^Zr]Zr-**3** and TCO–U36 was completed within 20 min and resulted in successful radiolabeling of TCO–U36 with high radiochemical yields regardless of the TCO-to-U36 ratio, demonstrating the suitability of the method for radiolabeling of antibodies with zirconium-89 in mild reaction conditions. When administered alone, the tetrazine [^89^Zr]Zr-**3** exhibited fast clearance and elimination mainly into urine, with only minor residual activity in the kidneys at 24 h p.i. in mice. [^89^Zr]Zr-**3** was successfully used for *in vivo* radiolabeling of the tumor antigen-bound U36 with a reasonable tumor uptake of 3.3 ± 0.5% ID/g when a high TCO-to-U36 ratio (27:1) was used in the antibody conjugation. However, the higher TCO-to-U36 ratio had its drawbacks as it significantly increased the liver accumulation of the U36 due to the altered pharmacokinetics of the functionalized antibody and increased the clearance from the blood. Decreasing the TCO-to-U36 ratio from 27:1 to 6:1 successfully reduced the unfavorable liver uptake by two-thirds but also resulted in lower tumor accumulation (1.5 ± 0.2% ID/g at 72 h). This may be explained by the lower IEDDA reaction efficiency at the lower TCO-to-U36 ratio. In pretargeted PET imaging applications, fast reaction kinetics at low concentrations are required for efficient *in vivo* labeling.^43^ The IEDDA reaction is characterized by the second-order reaction kinetics with dependence on concentration of the reactants, in our case, the TCO concentration at the target site. Decreasing the TCO-to-U36 ratio from 27:1 to 6:1 increased the tumor accumulation of the *in vitro*-radiolabeled U36 from 6.11 ± 1.12 to 17.1 ± 3.0% ID/g but resulted in a lower tumor accumulation in the pretargeted approach. Obviously, the 2.8 times higher antibody concentration in the tumor was not enough to compensate for the lower TCO-to-U36 ratio *in vivo*, resulting in lower TCO concentration in the tumor and consequently lower *in vivo* IEDDA reaction efficiency in the pretargeted approach. In addition, the higher TCO-mAb levels in blood were most probably contributed by consuming the [^89^Zr]Zr-**3** before it reached the tumor site.

Another explanation for the lower IEDDA reactivity could be the *in vivo* deactivation of TCO. Deactivation of TCO by isomerization in the presence of high thiol concentrations has been reported, leading to decreased *in vivo* reactivity and consequently lower tumor activities. Robillard et al. showed that in fresh mouse serum at 37 °C the *trans*-isomer converts into *cis*-cyclooctene with a half-life of 3.26 h. By attaching the TCO through a short linker, as done in this study, the deactivation half-life of TCO in circulation in mice was increased to 4 days.^44^ Indeed, we did not observe any statistically significant decrease in TCO reactivity between the groups that received [^89^Zr]Zr-**3** at 24 and 48 h p.i. when the lower TCO-to-U36 ratio was used. With the higher 27:1 TCO-to-U36 ratio, lower tumor activity was observed at the later time point, but this can be rather attributed to the altered pharmacokinetics of TCO–U36 at a high degree of conjugation than the *in vivo* isomerization of the TCO in this case.

*In vivo* IEDDA reaction yields can be improved by increasing the TCO concentration at the target site. However, as demonstrated by our results and reported previously by others, increasing the TCO-to-mAb conjugation ratio has its limitations since the pharmacokinetics of the antibody can be altered when too high conjugation ratios are used.^45,46^ When compared to the previous study with triazide-conjugated U36,^41^ the change in pharmacokinetics in the current study was mainly evidenced by the decreased tumor and blood radioactivity levels and increased liver uptake upon increasing the TCO-to-U36 ratio. This is most likely because of the increased lipophilicity of the antibody due to the conjugation.

The obtained results clearly demonstrate the potential and challenges of the pretargeted approach when utilizing IEDDA ligation between tetrazine and TCO. Clearance and metabolism of the tracer, the ratio between reactive TCO-to-antibody, and pharmacokinetics of the modified antibody all affect the *in vivo* labeling efficiency and the radioactivity accumulation into the tumor. The relatively long physical half-life of zirconium-89 allowed us to follow *in vitro*-labeled U36 for days and made it possible to make a direct comparison between the two different radiolabeling approaches. Even though the tumor accumulation of the *in vivo*-labeled U36 was lower than that of the *in vitro*-labeled U36, similar tumor-to-non-target tissue ratios were achieved due to the fast clearance of the tetrazine [^89^Zr]Zr-**3** (T/M ratios 23.49 ± 6.22 and 25.67 ± 6.30, respectively) but with significantly shorter radiation exposure time. The dosimetric calculations revealed significantly lower absorbed doses for the pretargeted approach, which demonstrates the dosimetric advantage of the pretargeted approach compared to that of the conventional direct antibody radiolabeling strategy even with the same radionuclide zirconium-89.

## Conclusions

Anti-CD44v6 monoclonal antibody U36 was successfully used for ^89^Zr-immuno-PET imaging of head-and-neck squamous cell carcinoma xenograft tumors using both a targeted and pretargeted approach. Our results demonstrate that the pretargeting of TCO–U36 with the tetrazine [^89^Zr]Zr-**3** constitutes a promising concept for *in vivo* pretargeted PET imaging on antibodies with zirconium-89 and warrants further investigation into radiolabeling of **3** with shorter-lived PET radionuclides like ^68^Ga. An alternative and potential method for *in vitro* radiolabeling of ^89^Zr-labeled radioimmunoconjugates is presented using IEDDA and [^89^Zr]Zr-**3**.

## Experimental Procedures

### Materials

All chemicals and solvents were obtained from commercial providers and were used without further purification. *N*-(4-(1,2,4,5-Tetrazin-3-yl)benzyl)-1-amino-3,6,9,12-tetraoxapentadecan-15-amide (Tz-PEG_5_-NHS) was purchased from Click Chemistry Tools (Scottsdale, AZ). *N*1-(5-Aminopentyl)-*N*1-hydroxy-*N*4-(5-(*N*-hydroxy-4-((5-(*N*-hydroxyacetamido)pentyl)amino)-4-oxobutanamido)pentyl)succinamide (DFO mesylate, **2**) was purchased from Merck, Darmstadt, Germany. *trans*-Cyclooctene-PEG_4_-NHS ester (TCO-NHS) was obtained from Jena Bioscience. Ultrapure water (18.2 MΩ) was prepared using a Milli-Q (mQ) Integral 10 water purification system. [^89^Zr]Zr-oxalate was purchased from Perkin Elmer and produced by BV Cyclotron VU, Amsterdam, The Netherlands. Two different HPLC systems and four different columns were used. A JASCO HPLC system with a Superdex 200 Increase 10/300 GL (300 × 10 mm, 8.6 μm) size exclusion column (GE Healthcare Life Sciences) was used, using 0.05 M phosphate buffer/0.15 M NaCl/0.01 NaN_3_ (pH 6.7) as an eluent (antibody analyses) and Grace, Alltima C18 (4.6 × 150 mm, 5 μm) with mQ/acetonitrile (ACN) (0.1% trifluoroacetic acid, TFA), ACN gradient 20–80%, 1 mL/min. A Shimadzu HPLC system with a Waters Symmetry Prep C18 (7.8 × 300 mm, 7 μm) was used, using 0.1% TFA in water/ACN as an eluent with ACN gradient 10–80%, 3 mL/min, UV detection at 270 nm and Phenomenex, Bio-Sep-SEC-s3060 (300 × 7.80 mm) with 0.05 M phosphate buffer/0.15 M NaCl (pH 6.7), 1 mL/min (DFO-PEG_5_-Tz purification). Iodogen tubes were acquired from Thermo Scientific Pierce (Iodination Tubes), Hampton, NH. ^1^H NMR and ^13^C NMR were measured with a Varian Mercury 300 MHz NMR equipment and time-of-flight electrospray ionization mass spectrometry (TOF-ESI-MS) mass spectrometry in a Bruker Daltonics micrOTOF Mass Spectrometer. MALDI measurements were done with a Bruker UltrafleXtreme 2 kHz MALDI-TOF/TOF Mass Spectrometer.

#### VU-SCC-OE Cell Line and Antibody U36

Monoclonal antibody, cmAb U36, targeting the head-and-neck squamous cell carcinoma (HNSCC) cell line VU-SCC-OE, binds to CD44v6 antigen of the tumor. The characteristics of the VU-SCC-OE cell line as well as the production and characterization of the mAb U36 have been described elsewhere.^31^

### Methods

#### Synthesis of *N*^1^-(4-(1,2,4,5-Tetrazin-3-yl)benzyl)-*N*^19^-(3,14,25-trihydroxy-2,10,13,21,24-pentaoxo-3,9,14,20,25-pentaazatriacontan-30-yl)-4,7,10,13,16-pentaoxanonadecanediamide (DFO-PEG_5_-Tz, **3**)

Compound **3** was synthesized from Tz-PEG_5_-NHS (**1**) (10–15 mg, 16.5–24.8 nmol, 1 equiv) and DFO mesylate (**2**) (18.4–27.6 mg, 24.8–37.2 nmol, 1.5 equiv) in 5 mL dimethyl formamide (DMF) using coupling reagents 1-[bis(dimethylamino)methylene]-1*H*-1,2,3-triazolo[4,5-*b*]pyridinium 3-oxide hexafluorophosphate (HATU) (12.6–18.9 mg, 33.1–49.7 nmol) and triethylamine (4.1–6.2 mg, 40.5–61.3 nmol) overnight at room temperature. The crude product was purified with a C18 SepPak light cartridge. The C18 cartridge was pretreated with 2 mL of EtOH and 10 mL of ultrapure water. Compound **3** was eluted using acetonitrile as an eluent, before the final purification step using semi-prep HPLC (Waters Symmetry Prep C18, ACN/mQ (0.1% TFA): gradient ACN 10–80%, 3 mL/mL) and evaporated to dryness. DFO-PEG_5_-Tz (3) was obtained as a pink solid with a 31 ± 11% yield (*n* = 3). The product was characterized by NMR and mass spectrometry: ^1^H NMR (300 MHz, CD_3_OD) δ 10.32 (3H, s), 8.56 (2H, d, *J* = 6.1 Hz), 7.60 (2H, d), 4.53 (2H, s), 3.79 (4H, t), 3.70 (3H, m), 3.59 (16H, m, *J* = 3.2), 3.16 (6H, m), 2.75 (6H, m), 2.55 (8H, t), 2.44 (4H, m), 2.08 (3H, s), 1.63 (6H, m), 1.51 (6H, m), 1.33 (6H, m); ^13^C NMR (75 MHz, CD_3_OD/D_2_O) δ 176.2, 176.1, 175.5, 175.0, 174.9, 168.4, 159.8, 134.4, 132.6, 130.2, 72.0, 69.0, 44.6, 41.1, 38.4, 32.4, 30.4, 29.7, 27.9, 25.5, 21.1. TOF-ESI-MS [M – H]^−^*m*/*z* calcd 1048.5757 for C_48_H_79_N_11_O_15_^–^, found 1048.5695.

#### Synthesis of [^89^Zr]Zr-DFO-PEG_5_-Tz ([^89^Zr]Zr-**3**)

[^89^Zr]Zr-oxalate in 1 M oxalic acid (5–100 MBq) was added to a glass vial followed by the addition of 1 M oxalic acid up to 200 μL total volume. Next, 90 μL of 2 M Na_2_CO_3_ was added and reacted for 3 min. Finally, DFO-PEG_5_-Tz (**3**) (1–100 μg, 0.94–94 nmol), diluted from a higher concentration, in 0.7–1.0 mL 0.5 M HEPES buffer (pH 7) was added to the mixture and the solution was incubated 20 min at room temperature. [^89^Zr]Zr-DFO-PEG_5_-Tz was purified with a C18 SepPak light cartridge using a 50% EtOH/saline solution as an eluent. The C18 cartridge was pretreated with 2 mL of EtOH and 10 mL of ultrapure water. The radiochemical purity was assessed with iTLC-SG (Agilent, Santa Clara) using 50 mM ethylenediaminetetraacetic acid (EDTA) as an eluent and with HPLC (Alltima C18 column, mQ/ACN with 0.1% TFA, ACN gradient 20–80%, 1 mL/min, *t*_R_ = 9.65 min). The radiolabeling yield was (80 ± 16%), and the radiochemical purity was >98%.

Stability of the radiolabeled [^89^Zr]Zr-**3** in formulation solution, diluted in 10% EtOH in saline + 0.1% Tween, 20 mM gentisic acid, pH = 5.2, was measured after 4, 24, and 48 h storage at °C, and stability was measured with iTLC-SG and HPLC (Alltima C18).

#### U36 Conjugation with TCO-PEG_4_-NHS

U36 (4 mg, 27 nmol) was conjugated with 10–40 equivalents (0.14–0.55 mg, 270–1080 nmol, 2.7–10.8 μL) of TCO-PEG_4_-NHS (in DMSO) in 1 mL PBS (pH adjusted to 8.5 with 0.1 M Na_2_CO_3_) at room temperature overnight. Conjugated U36 was purified with a PD-10 column and reconstituted to PBS (pH = 7) with an Amicon centrifugation filter (MWCO 10 kDa, 4000 G, 20 min). The TCO-to-U36 ratio was determined by matrix-assisted laser desorption/ionization-TOF-MS (MALDI-TOF-MS), calculating the mass difference of nonconjugated U36 to TCO-conjugated TCO–U36.

#### Synthesis of [^89^Zr]Zr-3–TCO–U36 (*In Vitro* Labeling)

TCO–U36 (0.5–1 mg, 3.4–6.8 nmol) and [^89^Zr]Zr-**3** (25–45 MBq, 3.5–7.0 μg, 3.4–6.8 nmol) were diluted in 0.5 mL of 0.5 M HEPES buffer, and the solution was shaken at room temperature for 20 min. ^89^Zr-labeled U36 was purified with a PD-10 column and concentrated with an Amicon centrifugation filter (MWCO 10 kDa, 4000 G, 20 min), and the purity of the product was confirmed by size exclusion HPLC (Superdex). The radiolabeling yield was 85 ± 4%, and the radiochemical purity was >99%.

#### Immunoreactivity of TCO–U36

Immunoreactivity of the TCO-conjugated U36 (27.2 TCO-to-U36) was analyzed with CD44v6-coated superparamagnetic immuno-beads. The binding experiment was done in triplicate with five bead concentrations (2.5 × 10^7^ to 1.6 × 10^6^ /mL) in a 1% bovine serum albumin (BSA) in PBS solution and in one control for nonspecific binding with a bead concentration of 1.6 × 10^6^ /mL, essentially as described by Lindmo et al.^47^ More detailed experimental conditions are described in the Supporting Information (SI).

#### Synthesis of [^125^I]I-U36

To an Iodogen tube (50 μg) (Thermo Fisher, Rockford, IL), 50 μL of 0.5 M NaH_2_PO_4_ (pH = 7.4), 344 μL of 0.1 M Na_2_HPO_4_, 125 μL U36 (0.6 mg, 3.98 nmol) in PBS, and 1 μL of ^125^I in 0.1 mM NaOH (19 MBq, 12.9 GBq/mL) were added, and the solution was gently shaken for 10 min, followed by the addition of 0.1 mL ascorbic acid (25 mg/mL) and 5 min shaking. The reaction mixture was transferred to a syringe connected to a filter (0.22 μm, Millex-GV, Millipore, Burlington, MS) followed by 0.4 mL of 0.1 M Na_2_HPO_4_ (pH = 6.8), used for an additional rinsing of the vial. The solution was filtered and purified on a PD-10 column with 0.9% NaCl/ascorbic acid (5 mg/mL, pH = 5) as an eluent (RCY = 18%, *n* = 1). Radiochemical purity was measured with SE-HPLC (Bio-Sep-SEC) resulting in >98% purity.

### Biological Evaluation

**Scheme 3:**
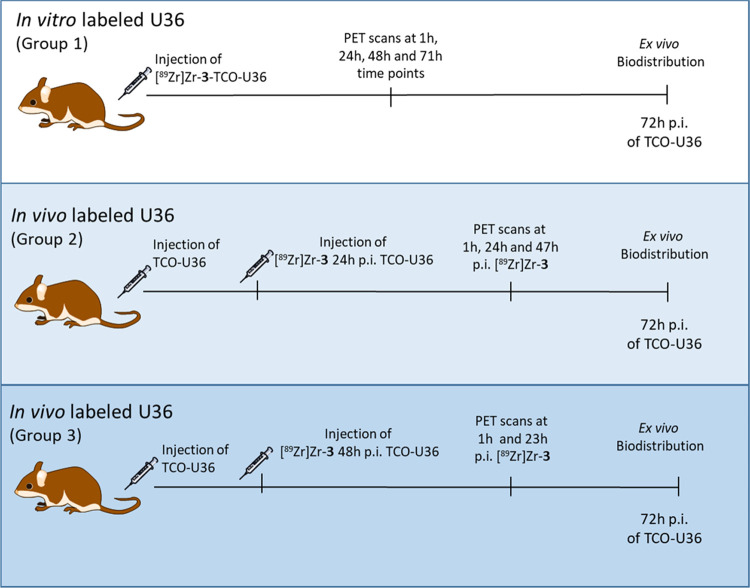
Experimental Scheme for the PET Imaging Studies

VU-SCC-OE cells (2 × 10^6^ cells/flank, volume: 100 μL/flank) were injected subcutaneously bilaterally (right and left flank). Experiments were performed according to the National Institute of Health principles of laboratory animal care and Dutch national law (“Wet op de proefdieren”. Stb 1985, 336) and a project license approved by the National Board of Animal Experimentation in Finland (ESAVI/12132/04.10.07/2017, approved on February 1st 2018) and in compliance with the respective institutional, national, and EU regulations and guidelines (Scheme 3).

#### Biodistribution Study of *In Vitro*-Labeled U36–TCO and *In Vivo* Labeling of U36–TCO with [^89^Zr]Zr-3 (27:1 TCO-to-U36)

Experiments were done in nude female mice (HSD:athymic nude *Foxn1*^*nu*^, 15–30 g; Charles River, Germany), aged 8–10 weeks at the time of the experiment, bearing subcutaneously implanted VU-SCC-OE xenografts (tumor volumes varied from 205 to 914 mm^3^). Mice were randomized to the three groups (*n* = 4/group): group 1 received the *in vitro*-labeled [^89^Zr]Zr-**3**–TCO–U36 and groups 2 and 3 for the pretargeted approach received [^89^Zr]Zr-**3** 24 and 48 h after U36–TCO administration. On day 1, group 1 mice were injected (i.v.) with *in vitro*-labeled [^89^Zr]Zr-**3**–TCO–U36 (4.4 ± 0.4 MBq, 0.1 mg, 0.66 nmol) and groups 2 and 3 mice were injected (i.v.) only with U36–TCO (0.1 mg, 0.66 nmol). For group 2, [^89^Zr]Zr-**3** (4.1 ± 0.3 MBq, 0.7 μmol, 0.66 nmol) was injected (i.v.) 24 h after U36–TCO injection and for group 3 (3.9 ± 0.5 MBq, 0.7 μmol, 0.66 nmol) (i.v.) 48 h after U36–TCO injection. Group 1 mice were imaged with PET-CT/MRI at 1 (dynamic scan), 24, 48, and 71 h after U36 injection, group 2 mice were imaged 1 (dynamic scan), 24, and 47 h, and group 3 were imaged 1 (dynamic scan) and 23 h after the injection of the tracer. All mice were sacrificed at 72 h p.i. of the U36 injection, and the collected organs (urine, blood, gall bladder, pancreas, spleen, kidney, liver, heart, lung, stomach, small intestine, large intestine + cecum, feces (1–2 pellets from the rectum), bladder, skeletal muscle, bone (tibia), bone (skull), brain, skin, and head) were weighted and the amount of radioactivity in each tissue was measured by a γ-counter. Radioactivity uptake was calculated as the percentage of the injected dose per gram of tissue (% ID/g). Quantitative PET image analysis was performed by defining regions of interest (ROIs) around the tumor with CT or MRI as the anatomical reference. Radioactivity concentration was expressed as an SUV, calculated using the average radioactivity concentration of the ROI normalized with the injected radioactivity dose and animal weight.

#### *Ex Vivo* Biodistribution of [^125^I]U36 and [^89^Zr]Zr-3–TCO–U36 Conjugates in Healthy Mice for Optimization of the TCO-to-cmAb Ratio

Biodistribution of the *in vitro*-labeled U36 with different TCO-to-U36 ratios and without TCO ([^125^I]U36) was investigated in healthy female nude mice (HSD:athymic nude *Foxn1*^*nu*^, 15–25 g, 8–10 weeks, (*n* = 4/group); Charles River, Germany). [^125^I]I-U36 (350 ± 50 kBq, 0.1 mg, 0.66 nmol) and [^89^Zr]Zr-**3**–TCO–U36 (150 ± 50 kBq, 0.1 mg, 0.66 nmol) with TCO-to-U36 ratios between 9:1 and 15:1 were injected i.v. (200 μL, saline). All mice were sacrificed at 72 h p.i., and the harvested organs (same as above) were weighed and the amount of radioactivity in each tissue was measured by a γ-counter. Radioactivity uptake was calculated as the percentage of the injected dose per gram of tissue (% ID/g).

#### Biodistribution Study of *In Vitro*-Labeled TCO–U36 and *In Vivo* Click Reaction (6:1 TCO-to-U36)

Experiments were done in nude female mice (HSD:athymic nude *Foxn1*^*nu*^, 15–30 g; Envigo, Horst, the Netherlands), aged 8–10 weeks at the time of the experiment, bearing subcutaneously implanted VU-SCC-OE xenografts (tumor volumes varied from 31 to 793 mm^3^). Mice were randomized to three groups as described above. At day 1, group 1 mice were injected (i.v.) with *in vitro*-labeled [^89^Zr]Zr-**3**–TCO–U36 (3.0 ± 0.3 MBq, 0.1 mg, 0.66 nmol) and groups 2 and 3 mice were injected (i.v.) only with TCO–U36 (0.1 mg, 0.66 nmol). For group 2, [^89^Zr]Zr-**3** (2.5 ± 0.2 MBq, 0.7 μmol, 0.66 nmol) was injected (i.v.) 24 h after the TCO–U36 injection and for group 3 (2.0 ± 0.2 MBq, 0.7 μmol, 0.66 nmol) (i.v.) 48 h after the U36–TCO injection. Group 1 mice were imaged with PET-CT at 1 (dynamic scan), 24, 48, and 71 h after cmAb injection, group 2 mice were imaged 1 (dynamic scan), 24, and 47 h, and group 3 were imaged 1 (dynamic scan) and 23 h after injection of the tracer. All mice were sacrificed at 72 h p.i. of U36, and the collected organs (same as above) were weighted and the amount of radioactivity in each tissue was measured by a γ-counter. Radioactivity uptake was calculated as the percentage of the injected dose per gram of tissue (% ID/g). Quantitative PET image analysis was performed by defining regions of interest (ROIs) around the tumor with CT as the anatomical reference. Radioactivity concentration was expressed as an SUV, calculated using the average radioactivity concentration of the ROI normalized with the injected radioactivity dose and animal weight.

#### Organ Dosimetry

The activity for each organ that was visible in PET/CT scans (heart, liver, lungs, spleen, kidneys, small intestine, large intestine, bladder, bone, and muscle) was determined using the mean activity concentration in VOIs with Vinci64 v 5.06 software. VOIs were independently drawn on all PET/CT scans for each mouse. The total activity in each organ was then calculated from the activity concentration and the Olinda 25 g mice model organ weight. Organ time–activity curves were created by collating the total activity from all mice fitted by exponential functions. Analytical integration of the fit provided the organ residence times, and this data was used as an input in OLINDA/EXM 2.1. This software was used for the calculation of organ-absorbed doses and the effective dose. Human dosimetry estimates were obtained from the residence times using OLINDA/EXM version 2.1 software with the adult model.

#### Statistics

The statistical difference was evaluated by Student’s *t*-test, where the significant probabilities were set at *p < 0.05, **p < 0.01, and ***p < 0.001.

## Supporting Information Available

Additional experimental details and results; method and result for immunoreactivity of U36–TCO; *ex vivo* biodistribution results from the animal experiments of [^89^Zr]Zr-**3** and U36–TCO with different TCO-to-cmAb ratios; NMR spectra of DFO-PEG_5_-Tz (**3**); and chromatograms from the different stages of [^89^Zr]Zr-**3** and [^89^Zr]Zr-**3**–TCO–U36 syntheses (PDF)

## References

[ref1] Lamberts L. E.; Williams S. P.; Terwisscha van Scheltinga A.; Lub-de Hooge M. N.; Schroeder C. P.; Gietema J. A.; Brouwers A. H.; De Vries E. Antibody positron emission tomography imaging in anticancer drug development. J. Clin. Oncol. 2015, 33, 1491–1504. 10.1200/JCO.2014.57.8278. 25779566

[ref2] Van Dongen G.; Huisman M.; Boellaard R.; Hendrikse N. H.; Windhorst A.; Visser G.; Molthoff C.; Vugts D. 89Zr-immuno-PET for imaging of long circulating drugs and disease targets: why, how and when to be applied. Q. J. Nucl. Med. Mol. Imaging 2015, 59, 18–38. 25517081

[ref3] Yaghoubi S.; Karimi M. H.; Lotfinia M.; Gharibi T.; Mahi-Birjand M.; Mahi-Birjand M.; Kavi E.; Hosseini F.; Sineh Sepehr K.; Khatami M.; Bagheri N.; et al. Potential drugs used in the antibody–drug conjugate (ADC) architecture for cancer therapy. J. Cell. Physiol. 2020, 235, 31–64. 10.1002/jcp.28967. 31215038

[ref4] Harsini S.; Rezaei N. Cancer Imaging with Radiolabeled Monoclonal Antibodies. In Cancer Immunology; Springer, 2020; pp 739–760.

[ref5] Bouchard H.; Viskov C.; Garcia-Echeverria C. Antibody–drug conjugates—a new wave of cancer drugs. Bioorg. Med. Chem. Lett. 2014, 24, 5357–5363. 10.1016/j.bmcl.2014.10.021. 25455482

[ref6] Beck A.; Goetsch L.; Dumontet C.; Corvaïa N. Strategies and challenges for the next generation of antibody–drug conjugates. Nat. Rev. Drug Discovery 2017, 16, 315–337. 10.1038/nrd.2016.268. 28303026

[ref7] Klein C.; Schaefer W.; Regula J. T. The Use of CrossMAb Technology for the Generation of Bi-and Multispecific Antibodies. In MAbs; Taylor & Francis, 2016. 10.1080/19420862.2016.1197457 PMC4968094 27285945

[ref8] Mastrangeli R.; Palinsky W.; Bierau H. Glycoengineered antibodies: towards the next-generation of immunotherapeutics. Glycobiology 2019, 29, 199–210. 10.1093/glycob/cwy092. 30289453

[ref9] Freise A. C.; Wu A. M. In vivo imaging with antibodies and engineered fragments. Mol. Immunol. 2015, 67, 142–152. 10.1016/j.molimm.2015.04.001. 25934435 PMC4529772

[ref10] Van Dongen G. A.; Visser G. W.; Lub-de Hooge M. N.; De Vries E. G.; Perk L. R. Immuno-PET: a navigator in monoclonal antibody development and applications. Oncologist 2007, 12, 1379–1389. 10.1634/theoncologist.12-12-1379. 18165614

[ref11] Aluicio-Sarduy E.; Ellison P. A.; Barnhart T. E.; Cai W.; Nickles R. J.; Engle J. W. PET radiometals for antibody labeling. J. Labelled Compd. Radiopharm. 2018, 61, 636–651. 10.1002/jlcr.3607. PMC6050152 29341227

[ref12] Stéen E. J. L.; Edem P. E.; Nørregaard K.; Jørgensen J. T.; Shalgunov V.; Kjaer A.; Herth M. M. Pretargeting in nuclear imaging and radionuclide therapy: Improving efficacy of theranostics and nanomedicines. Biomaterials 2018, 179, 209–245. 10.1016/j.biomaterials.2018.06.021. 30007471

[ref13] Nayak T. K.; Brechbiel M. W. Radioimmunoimaging with Longer-Lived Positron-Emitting Radionuclides: Potentials and Challenges. Bioconjugate Chem. 2009, 20, 825–841. 10.1021/bc800299f. PMC3397469 19125647

[ref14] Wu A. M. Antibodies and Antimatter: The Resurgence of Immuno-PET. J. Nucl. Med. 2009, 50, 2–5. 10.2967/jnumed.108.056887. 19091888

[ref15] Goldenberg D. M.; Sharkey R. M.; Paganelli G.; Barbet J.; Chatal J.-F. Antibody pretargeting advances cancer radioimmunodetection and radioimmunotherapy. J. Clin. Oncol. 2006, 24, 816 10.1200/JCO.2005.03.8471. 16380412

[ref16] Rossin R.; Läppchen T.; van den Bosch S. M.; Laforest R.; Robillard M. S. Diels–Alder Reaction for Tumor Pretargeting: In Vivo Chemistry Can Boost Tumor Radiation Dose Compared with Directly Labeled Antibody. J. Nucl. Med. 2013, 54, 1989–1995. 10.2967/jnumed.113.123745. 24092936

[ref17] Meyer J.-P.; Houghton J. L.; Kozlowski P.; Abdel-Atti D.; Reiner T.; Pillarsetty N.V.K.; Scholz W. W.; Zeglis B. M.; Lewis J. S. 18F-Based Pretargeted PET Imaging Based on Bioorthogonal Diels-Alder Click Chemistry. Bioconjugate Chem. 2016, 27, 298–301. 10.1021/acs.bioconjchem.5b00504. PMC4759614 26479967

[ref18] Rossin R.; Renart Verkerk P.; van den Bosch S. M.; Vulders R.C.M.; Verel I.; Lub J.; Robillard M. S. In Vivo Chemistry for Pretargeted Tumor Imaging in Live Mice. Angew. Chem. 2010, 49, 3375–3378. 10.1002/anie.200906294. 20391522

[ref19] Läppchen T.; Rossin R.; van Mourik T. R.; Gruntz G.; Hoeben F.J.M.; Versteegen R. M.; Janssen H. M.; Lub J.; Robillard M. S. DOTA-tetrazine probes with modified linkers for tumor pretargeting. Nucl. Med. Biol. 2017, 55, 19–26. 10.1016/j.nucmedbio.2017.09.001. 29028502

[ref20] Zeglis B. M.; Mohindra P.; Weissmann G. I.; Divilov V.; Hilderbrand S. A.; Weissleder R.; Lewis J. S. Modular strategy for the construction of radiometalated antibodies for positron emission tomography based on inverse electron demand diels–alder click chemistry. Bioconjugate Chem. 2011, 22, 2048–2059. 10.1021/bc200288d. PMC3197258 21877749

[ref21] Meimetis L. G.; Boros E.; Carlson J. C.; Ran C.; Caravan P.; Weissleder R. Bioorthogonal Fluorophore Linked DFO— Technology Enabling Facile Chelator Quantification and Multimodal Imaging of Antibodies. Bioconjugate Chem. 2016, 27, 257–263. 10.1021/acs.bioconjchem.5b00630. PMC4858350 26684717

[ref22] Damerow H.; Hübner R.; Judmann B.; Schirrmacher R.; Wängler B.; Fricker G.; Wängler C. Side-by-Side Comparison of Five Chelators for 89Zr-Labeling of Biomolecules: Investigation of Chemical/Radiochemical Properties and Complex Stability. Cancers 2021, 13, 6349 10.3390/cancers13246349. 34944969 PMC8699488

[ref23] Edem P. E.; Jørgensen J. T.; Nørregaard K.; Rossin R.; Yazdani A.; Valliant J. F.; Robillard M.; Herth M. M.; Kjaer A. Evaluation of a 68Ga-Labeled DOTA-Tetrazine as a PET Alternative to 111In-SPECT Pretargeted Imaging. Molecules 2020, 25, 463 10.3390/molecules25030463. PMC7036891 31979070

[ref24] Houghton J. L.; Membreno R.; Abdel-Atti D.; Cunanan K. M.; Carlin S.; Scholz W. W.; Zanzonico P. B.; Lewis J. S.; Zeglis B. M. Establishment of the in vivo efficacy of pretargeted radioimmunotherapy utilizing inverse electron demand Diels-Alder click chemistry. Mol. Cancer Ther. 2017, 16, 124–133. 10.1158/1535-7163.MCT-16-0503. 28062708 PMC5221649

[ref25] Rondon A.; Schmitt S.; Briat A.; Ty N.; Maigne L.; Quintana M.; Membreno R.; Zeglis B. M.; Navarro-Teulon I.; Pouget J.-P.; et al. Pretargeted radioimmunotherapy and SPECT imaging of peritoneal carcinomatosis using bioorthogonal click chemistry: probe selection and first proof-of-concept. Theranostics 2019, 9, 6706 10.7150/thno.35461. 31588245 PMC6771248

[ref26] Houghton J. L.; Zeglis B. M.; Abdel-Atti D.; Sawada R.; Scholz W. W.; Lewis J. S. Pretargeted Immuno-PET of Pancreatic Cancer: Overcoming Circulating Antigen and Internalized Antibody to Reduce Radiation Doses. J. Nucl. Med. 2016, 57, 453–459. 10.2967/jnumed.115.163824. 26471693 PMC4852470

[ref27] Cook B. E.; Adumeau P.; Membreno R.; Carnazza K. E.; Brand C.; Reiner T.; Agnew B. J.; Lewis J. S.; Zeglis B. M. Pretargeted PET Imaging Using a Site-Specifically Labeled Immunoconjugate. Bioconjugate Chem. 2016, 27, 1789–1795. 10.1021/acs.bioconjchem.6b00235. PMC5102008 27356886

[ref28] Meyer J.-P.; Adumeau P.; Lewis J. S.; Zeglis B. M. Click Chemistry and Radiochemistry: The First 10 Years. Bioconjugate Chem. 2016, 27, 2791–2807. 10.1021/acs.bioconjchem.6b00561. PMC5193009 27787983

[ref29] Altai M.; Membreno R.; Cook B.; Tolmachev V.; Zeglis B. M. Pretargeted Imaging and Therapy. J. Nucl. Med. 2017, 58, 1553–1559. 10.2967/jnumed.117.189944. 28687600 PMC5632733

[ref30] Zeglis B. M.; Sevak K. K.; Reiner T.; Mohindra P.; Carlin S. D.; Zanzonico P.; Weissleder R.; Lewis J. S. A Pretargeted PET Imaging Strategy Based on Bioorthogonal Diels–Alder Click Chemistry. J. Nucl. Med. 2013, 54, 1389–1396. 10.2967/jnumed.112.115840. 23708196 PMC4151562

[ref31] Schrijvers A. H. G. J.; Quak J. J.; Uyterlinde A. M.; van Walsum M.; Meijer C. J. L. M.; Snow G. B.; van Dongen G. A. M. S. MAb U36, a Novel Monoclonal Antibody Successful in Immunotargeting of Squamous Cell Carcinoma of the Head and Neck. Cancer Res. 1993, 53, 4383–4390. 8364934

[ref32] Leung K. 89Zr-N-Succinyldesferal-Anti-CD44v6 Chimeric Monoclonal Antibody U36; Molecular Imaging and Contrast Agent Database, 2004. 20641988

[ref33] Bree R. D.; Roos J.; Quak J.; Den Hollander W.; Snow G.; Van Dongen G. Clinical screening of monoclonal antibodies 323/A3, cSF-25 and K928 for suitability of targetting tumours in the upper aerodigestive and respiratory tract. Nucl. Med. Commun. 1994, 15, 613–627. 10.1097/00006231-199408000-00006. 7970443

[ref34] Van Hal N. L.; Van Dongen G. A.; Rood-Knippels E. M.; Van Der Valk P.; Snow G. B.; Brakenhoff R. H. Monoclonal antibody U36, a suitable candidate for clinical immunotherapy of squamous-cell carcinoma, recognizes a CD44 isoform. Int. J. Cancer 1996, 68, 520–527. 10.1002/(SICI)1097-0215(19961115)68:4<520::AID-IJC19>3.0.CO;2-8. 8945625

[ref35] Heider K.-H.; Kuthan H.; Stehle G.; Munzert G. CD44v6: a target for antibody-based cancer therapy. Cancer Immunol. Immunother. 2004, 53, 567–579. 10.1007/s00262-003-0494-4. 14762695 PMC11032850

[ref36] Zhou D.-x.; Liu Y.-x.; Xue Y.-h. Expression of CD44v6 and its association with prognosis in epithelial ovarian carcinomas. Pathol. Res. Int. 2012, 2012, 908206 10.1155/2012/908206. PMC3317067 22482084

[ref37] Nestor M.; Andersson K.; Lundqvist H. Characterization of 111In and 177Lu-labeled antibodies binding to CD44v6 using a novel automated radioimmunoassay. J. Mol. Recognit. 2008, 21, 179–183. 10.1002/jmr.883. 18438972

[ref38] Sandström K.; Haylock A.-K.; Spiegelberg D.; Qvarnström F.; Wester K.; Nestor M. A novel CD44v6 targeting antibody fragment with improved tumor-to-blood ratio. Int. J. Oncol. 2012, 40, 1525–1532. 10.3892/ijo.2012.1352. 22307465

[ref39] Verel I.; Heider K. H.; Siegmund M.; Ostermann E.; Patzelt E.; Sproll M.; Snow G. B.; Adolf G. R.; Van Dongen G. A. Tumor targeting properties of monoclonal antibodies with different affinity for target antigen CD44V6 in nude mice bearing head-and-neck cancer xenografts. Int. J. Cancer 2002, 99, 396–402. 10.1002/ijc.10369. 11992408

[ref40] Börjesson P. K.; Jauw Y. W.; Boellaard R.; De Bree R.; Comans E. F.; Roos J. C.; Castelijns J. A.; Vosjan M. J.; Kummer J. A.; Leemans C. R.; et al. Performance of immuno–positron emission tomography with zirconium-89-labeled chimeric monoclonal antibody U36 in the detection of lymph node metastases in head and neck cancer patients. Clin. Cancer Res. 2006, 12, 2133–2140. 10.1158/1078-0432.CCR-05-2137. 16609026

[ref41] Vugts D. J.; Vervoort A.; Stigter-van Walsum M.; Visser G. W. M.; Robillard M. S.; Versteegen R. M.; Vulders R. C. M.; Herscheid J.D.M.; van Dongen G. A. M. S. Synthesis of Phosphine and Antibody–Azide Probes for in Vivo Staudinger Ligation in a Pretargeted Imaging and Therapy Approach. Bioconjugate Chem. 2011, 22, 2072–2081. 10.1021/bc200298v. 21854058

[ref42] Börjesson P. K.; Jauw Y. W.; de Bree R.; Roos J. C.; Castelijns J. A.; Leemans C. R.; van Dongen G. A.; Boellaard R. Radiation dosimetry of 89Zr-labeled chimeric monoclonal antibody U36 as used for immuno-PET in head and neck cancer patients. J. Nucl. Med. 2009, 50, 1828–1836. 10.2967/jnumed.109.065862. 19837762

[ref43] Oliveira B. L.; Guo Z.; Bernardes G. Inverse electron demand Diels–Alder reactions in chemical biology. Chem. Soc. Rev. 2017, 46, 4895–4950. 10.1039/C7CS00184C. 28660957

[ref44] Rossin R.; van den Bosch S. M.; ten Hoeve W.; Carvelli M.; Versteegen R. M.; Lub J.; Robillard M. S. Highly Reactive trans-Cyclooctene Tags with Improved Stability for Diels–Alder Chemistry in Living Systems. Bioconjugate Chem. 2013, 24, 1210–1217. 10.1021/bc400153y. 23725393

[ref45] Hamblett K. J.; Senter P. D.; Chace D. F.; Sun M. M.; Lenox J.; Cerveny C. G.; Kissler K. M.; Bernhardt S. X.; Kopcha A. K.; Zabinski R. F.; et al. Effects of drug loading on the antitumor activity of a monoclonal antibody drug conjugate. Clin. Cancer Res. 2004, 10, 7063–7070. 10.1158/1078-0432.CCR-04-0789. 15501986

[ref46] Ryman J. T.; Meibohm B. Pharmacokinetics of monoclonal antibodies. CPT: Pharmacometrics Syst. Pharmacol. 2017, 6, 576–588. 10.1002/psp4.12224. 28653357 PMC5613179

[ref47] Lindmo T.; Boven E.; Cuttitta F.; Fedorko J.; Bunn P. Jr Determination of the immunoreactive function of radiolabeled monoclonal antibodies by linear extrapolation to binding at infinite antigen excess. J. Immunol. Methods 1984, 72, 77–89. 10.1016/0022-1759(84)90435-6. 6086763

