# Supplementary material for: Pretargeted PET Imaging with a TCO-Conjugated Anti-CD44v6 Chimeric mAb U36 and [89Zr]Zr-DFO-PEG5-Tz
Source: Bioconjug Chem. 2022 Apr 20;33(5):956–68. doi: 10.1021/acs.bioconjchem.2c00164 (PMC9121349; doi:10.1021/acs.bioconjchem.2c00164)
Supplement: Supplementary file 1 — bc2c00164_si_001.pdf [file bc2c00164_si_001.pdf]

Pretargeted PET imaging with a TCO-conjugated anti-CD44v6 chimeric mAb U36 and [^{89}Zr]Zr-DFO-PEG$_5$-Tz

Dave Lumen[1], Danielle Vugts[2*], Marion Chomet[2], Surachet Imlimthan[1], Mirkka Sarparanta[1], Ricardo Vos[2], Maxime Schreurs[2], Mariska Verlaan[2], Pauline Lang[1,3], Eero Hippeläinen[4], Wissam Beaino[2], Albert D. Windhorst[2], Anu J. Airaksinen[1,5*]

1 Department of Chemistry, Radiochemistry, University of Helsinki, FI-00014 Helsinki, Finland
2 Amsterdam UMC, Vrije Universiteit Amsterdam, Radiology & Nuclear Medicine, Cancer Center Amsterdam, De Boelelaan 1117, 1081 HV Amsterdam, The Netherlands
3 Current address: Chemistry Research Laboratory, Department of Chemistry, University of Oxford, 12MansfieldRoad, Oxford, OX13TA, United Kingdom
4 HUS Medical Imaging Center, Clinical Physiology and Nuclear Medicine, University of Helsinki and Helsinki University Hospital, 00029 HUS, Helsinki, Finland
5 Turku PET Centre, Department of Chemistry, University of Turku, 20520 Turku, Finland

* Correspondence: d.vugts@amsterdamumc.nl, anu.airaksinen@utu.fi

Materials and methods

VU-SCC-OE Cells. VU-SCC-OE cells were cultured in high glucose (4.5g l^{-1}) DMEM culture medium, supplemented with 10% FBS, 1% L-glutamine, penicillin (100 IU ml^{-1}) and streptomycin (100 µg ml^{-1}). All the cells were cultured in the 5% CO_2 -incubator at 37 °C, and 95% relative humidity. The culture media were changed every other day. Prior to the test, 0.04% EDTA in PBS was used to detach the cells because the trypsinization leads to loss of the antigenicity of the U36 antigen.

Immunoreactivity of [^{89}Zr]Zr-3-TCO-U36.

Immunoreactivity of the TCO conjugated mAb (27:1 TCO-to-cmAb) was analyzed with a fusion protein GST-CD44v6 (Boehringer Ingelheim Austria GmbH, Vienna) coated Dynabeads™ M-280 Tosylactivated (ThermoFisher Scientific).VU-SCC-OE cells were not used for the immunoreactivity test, because CD44v6 is easily cleaved from the surface of the cells during the cell culturing procedures. The binding experiment was carried out in triplicate with five bead concentrations (2.5×10^7-1.6×10^6 /ml) in a 1 % BSA in PBS solution and in one control for non-specific binding with a bead concentration of 1.6×10^6 /ml. 0.02 µg [^{89}Zr]Zr-**3**-TCO-U36 (100–200 kBq) was added to each sample and for the determination of non-specific binding, 10 µg excess of the unlabeled antibody was added to the control sample. Samples were incubated and shaken for 1 h at 37 °C before changing to 4 °C for 10 min. Samples were centrifuged at 2000 rpm for 5 min. Supernatant was separated from the pellets and both were measured with a gamma counter.

Animal experiments.

Ex vivo biodistribution study of [^{89}Zr]Zr-DFO-PEG$_5$-Tz ([^{89}Zr]Zr-3). Biodistribution of [^{89}Zr]Zr-**3** was investigated in healthy female nude mice (HSD:Athymic nude Foxn1$^{nu/nu}$, weighing 15–30 g, aged 8–10 weeks; Charles River, Germany) at 1 h, 4 h and 24 h and only for 24 h in nude mice bearing subcutaneously implanted VU-SCC-OE xenografts. [^{89}Zr]Zr-**3** (350±50 kBq, 0.7 µmol, 0.66 nmol)

in 100 µl of 10% EtOH in saline (0.1% Tween, 20 mM gentisic acid), was injected to mice (n=16) (r.o.) and mice were sacrificed at 1 h (n=4) , 4 h (n=4) and 24 h (n=8) p.i. and the harvested organs (urine, blood, gall bladder, pancreas, spleen, kidney, liver, heart, lung, stomach, small intestine, large intestine + cecum, feces (1-2 pellets from the rectum), bladder, skeletal muscle, bone (thigh), bone (skull), brain, skin, tumor and head) were weighted and the amount of radioactivity in each tissue was measured by a γ-counter. Radioactivity uptake was calculated as the percentage of the injected dose per gram of tissue (%ID/g).

Results and conclusions

Immunoreactivity of U36-TCO (27:1 TCO-to-U36) with CD44v6-coated immunobeads (Figure S1). The analysis shows the immunoreactivity response of the TCO-conjugated antibody with increasing antigen concentration. Even though 100 % immunoreactivity was not reached, results still indicate high immunoreactivity for conjugated particles with a maximum of 91.6±1.3% binding corrected to non-specific binding.

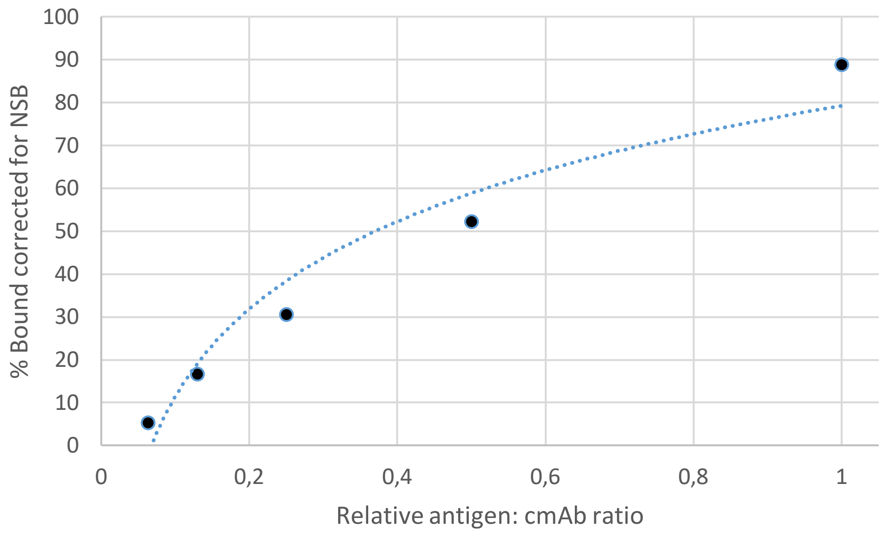

Figure S1. Immunoreactivity binding plots of U36-TCO (27:1 TCO-to-cmAb) with different CD44v6-bead-concentrations (2.5×10^{7}–1.6×10^{6} /ml). The binding experiment was carried out in triplicate and the results are presented as mean ± SD.

***Ex vivo* biodistribution study of [^{89}Zr]Zr-DFO-PEG$_5$-Tz ([^{89}Zr]Zr-3).** [^{89}Zr]Zr-**3** exhibited favorable pharmacokinetics with fast clearance with elimination mainly via kidneys to urine (572±264 %ID/g in urine at 1 h p.i.) with only low level of residual radioactivity in blood, liver and kidney at 1 h p.i. (0.48±0.06 %ID/g, 1.73±0.53 %ID/g and 3.49±0.28 %ID/g, respectively) (Figure S2).

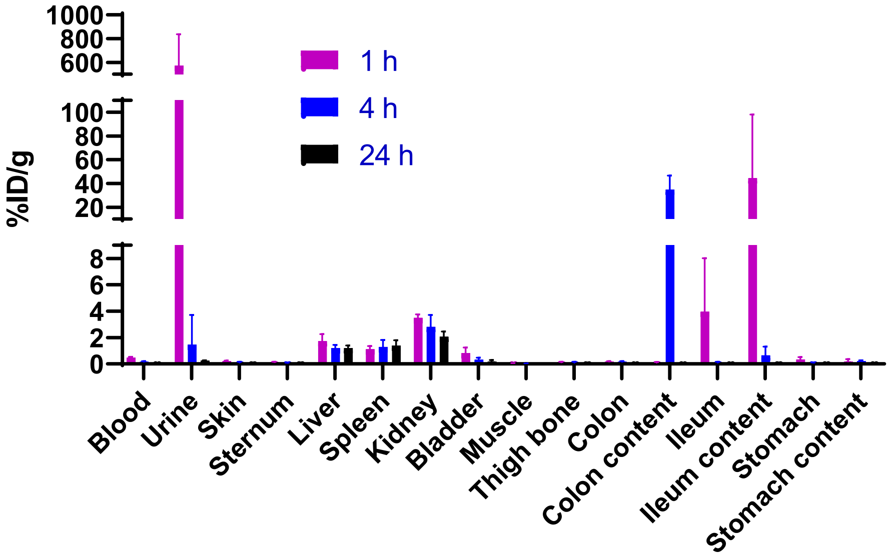

Figure S2. *Ex vivo* biodistribution of [^{89}Zr]Zr-**3** at 1 h, 4 h and 24 h time points in athymic nude mice. The results are presented as %ID/g ± SD, n = 4.

Table S1. *Ex vivo* biodistribution of [^{89}Zr]Zr-**3** in athymic nude mice at 1 h, 4 h and 24 h timepoints. The results are presented as %ID/g ± SD, n = 4.

	**[^{89}Zr]Zr-3 1 h**	**[^{89}Zr]Zr-3 4 h**	**[^{89}Zr]Zr-3 24 h**
**TISSUE**	**%ID/g**	**%ID/g**	**%ID/g**
**Blood**	0.48 ± 0.06	0.17 ± 0.04	0.04 ± 0.01
**Urine (%ID)**	31.38 ± 23.0	0.22 ± 0.40	0.02 ± 0.02
**Skin**	0.22 ± 0.03	0.11 ± 0.05	0.07 ± 0.02
**Sternum**	0.14 ± 0.03	0.08 ± 0.03	0.07 ± 0.03
**Heart**	0.15 ± 0.04	0.06 ± 0.02	0.04 ± 0.01
**Lung**	0.67 ± 0.10	0.37 ± 0.03	0.27 ± 0.20
**Liver**	1.73 ± 0.53	1.21 ± 0.23	1.21 ± 0.19
**Spleen**	1.11 ± 0.24	1.29 ± 0.52	1.40 ± 0.39
**Kidney**	3.49 ± 0.28	2.82 ± 0.90	2.06 ± 0.39
**Bladder**	0.82 ± 0.42	0.32 ± 0.15	0.19 ± 0.11
**Muscle**	0.05 ± 0.01	0.02 ± 0.01	0.01 ± 0.00
**Thigh bone**	0.12 ± 0.04	0.11 ± 0.05	0.08 ± 0.03
**Colon**	0.18 ± 0.02	0.16 ± 0.04	0.06 ± 0.02
**Ileum**	3.95 ± 4.06	0.11 ± 0.06	0.03 ± 0.00
**Stomach**	0.34 ± 0.18	0.10 ± 0.03	0.06 ± 0.01

Table S2. Ex vivo biodistribution of *in vitro* and *in vivo* radiolabeld TCO-U36 (TCO-to-cmAb: 27:1) and [^{89}Zr]Zr-**3** (at 24 h time point) in VU-SCC-OE xenografted mice at 72 h timepoint. The results are presented as %ID/g ± SD, n = 4.

	In vitro labeled [^{89}Zr]Zr-3-TCO-U36	[^{89}Zr]Zr-3 24 h p.i. of U36-TCO	[^{89}Zr]Zr-3 48 h p.i. of U36-TCO	[^{89}Zr]Zr-3 24 h
**TISSUE**	**%ID/g**	**%ID/g**	**%ID/g**	**%ID/g**
**Blood**	2.77 ± 1.37	2.88 ± 0.45	1.34 ± 1.08	0.04 ± 0.02
**Urine (%ID)**	0.10 ± 0.06	0.08 ± 0.05	0.03 ± 0.02	0.02 ± 0.01
**Tumor**	6.11 ± 1.12	3.31 ± 0.54	1.45 ± 0.62	0.07 ± 0.12
**Skin**	2.77 ± 0.32	1.40 ± 0.63	0.63 ± 0.30	0.08 ± 0.03
**Sternum**	1.09 ± 0.36	0.52 ± 0.16	0.28 ± 0.11	0.05 ± 0.02
**Heart**	1.22 ± 0.30	0.93 ± 0.11	0.45 ± 0.20	0.05 ± 0.02
**Lung**	1.45 ± 0.40	1.29 ± 0.17	0.68 ± 0.34	0.10 ± 0.01
**Liver**	14.14 ± 2.88	3.85 ± 1.48	1.33 ± 0.36	0.47 ± 0.24
**Spleen**	3.70 ± 1.13	1.27 ± 0.22	0.92 ± 0.50	0.07 ± 0.02
**Kidney**	3.97 ± 0.52	4.48 ± 0.63	6.25 ± 1.67	4.01 ± 1.90
**Bladder**	1.78 ± 0.51	1.08 ± 0.25	0.63 ± 0.25	0.16 ± 0.06
**Muscle**	0.28 ± 0.06	0.25 ± 0.09	0.11 ± 0.06	0.02 ± 0.01
**Thigh bone**	1.31 ± 0.49	0.53 ± 0.09	0.37 ± 0.18	0.06 ± 0.02
**Colon**	0.84 ± 0.38	0.45 ± 0.09	0.34 ± 0.12	0.07 ± 0.02
**Ileum**	1.51 ± 0.76	0.53 ± 0.07	0.68 ± 0.47	0.05 ± 0.03
**Stomach**	0.80 ± 0.18	0.46 ± 0.08	0.34 ± 0.13	0.06 ± 0.02

***Ex vivo* biodistribution of pretargeted TCO-U36 with 6:1 TCO-to-cmAb ratio traced with [^{89}Zr]Zr-3 24 h or 48 h after the cmAb injection.** For the *in vivo* labeled antibodies, the *ex vivo* biodistribution pattern was similar at 72 h p.i. of the cmAb, regardless of the tracer injection time (24 h and 48 h p.i. cmAb) (Figure S3). Liver and spleen values were three times lower compared to the tumor values, but blood radioactivity levels were still higher which could increase the tumor values at later time point and thus the target to non-target ratios.

***In vivo* radiolabeling efficiency of TCO-U36**. Calculation of the in vivo radiolabeling efficiency is based on an assumption that [^{89}Zr]Zr-**3**-TCO-U36 and TCO-U36 have similar biodistribution and pharmacokinetics when the TCO conjugation rate of the cmAb is equal. Based on this assumption, [^{89}Zr]Zr-**3**-TCO-U36 was used for calculating expected amount of TCO-U36 in the tumor at certain time point (i.e. the max. achievable %ID/g of radioactivity in the tumor). In vivo radiolabeling efficiency of mAb in the tumor was then calculated based on the injected [^{89}Zr]Zr-**3** radioactivity, while keeping the molar ratio between [^{89}Zr]Zr-**3** and mAb constant (1:1).

Table S3. Ex vivo biodistribution values for *in vitro* and *in vivo* radiolabeld TCO-U36 (6:1TCO-to-cmAb) in VU-SCC-OE xenografted mice at 72 h timepoint. The results are presented as %ID/g ± SD, n = 4.

	In vitro labeled [^{89}Zr]Zr-3-TCO-U36	[^{89}Zr]Zr-3 24 h p.i. of U36-TCO	[^{89}Zr]Zr-3 48 h p.i. of U36-TCO
**TISSUE**	**%ID/g**	**%ID/g**	**%ID/g**
**Blood**	7.37 ± 2.93	0.78 ± 0.17	0.94 ± 0.35
**Urine (%ID)**	0.29 ± 0.31	0.09 ± 0.16	0.02 ± 0.01
**Tumor**	17.14 ± 2.95	1.58 ± 0.29	1.53 ± 0.23
**Skin**	3.45 ± 0.71	0.35 ± 0.05	0.43 ± 0.06
**Sternum**	2.37 ± 0.31	0.29 ± 0.05	0.38 ± 0.06
**Heart**	1.96 ± 0.69	0.20 ± 0.05	0.28 ± 0.07
**Lung**	3.42 ± 1.18	0.40 ± 0.07	0.48 ± 0.16
**Liver**	5.47 ± 1.08	0.41 ± 0.10	0.53 ± 0.05
**Spleen**	3.45 ± 1.52	0.30 ± 0.05	0.29 ± 0.15
**Kidney**	4.49 ± 0.48	0.97 ± 0.27	1.00 ± 0.66
**Bladder**	2.78 ± 0.51	0.40 ± 0.09	0.40 ± 0.08
**Muscle**	0.67 ± 0.08	0.07 ± 0.01	0.10 ± 0.03
**Thigh bone**	3.78 ± 1.11	0.54 ± 0.19	0.65 ± 0.22
**Colon**	1.01 ± 0.33	0.14 ± 0.05	0.12 ± 0.04
**Ileum**	1.92 ± 1.50	0.26 ± 0.06	0.25 ± 0.14
**Stomach**	1.02 ± 0.30	0.13 ± 0.03	0.15 ± 0.02

Table S4. Volume (mm^3) and %ID values from image analysis at 71 h time point when 6:1 TCO-to-cmAb ratio was used. Calculated %ID/V values demonstrate that small tumors (< 100 mm^3) had clearly higher activity concentrations compared to the bigger ones.

**24 h p.i. U36**	Mouse 1		Mouse 2		Mouse 3		Mouse 4	
**V (mm3)**	420	423	593	489	49	478	329	262
**%ID**	1.33	1.30	1.39	1.27	0.66	1.56	1.34	1.17
**%ID/V**	0.003	0.003	0.002	0.003	0.013	0.003	0.004	0.004
**48 h p.i. U36**	Mouse 1		Mouse 2		Mouse 3		Mouse 4	
**V (mm3)**	443	294	193	242	31	314	280	309
**%ID**	0.86	0.90	0.66	0.62	0.37	0.91	1.01	1.17
**%ID/V**	0.002	0.003	0.003	0.003	0.012	0.003	0.004	0.004
**In vitro labeled**	Mouse 1		Mouse 2		Mouse 3		Mouse 4	
**V (mm3)**	207	793	722	47	496	97	167	603
**%ID**	7.36	12.15	14.53	5.61	9.34	8.91	8.21	11.02
**%ID/V**	0.036	0.015	0.020	0.120	0.019	0.092	0.037	0.018

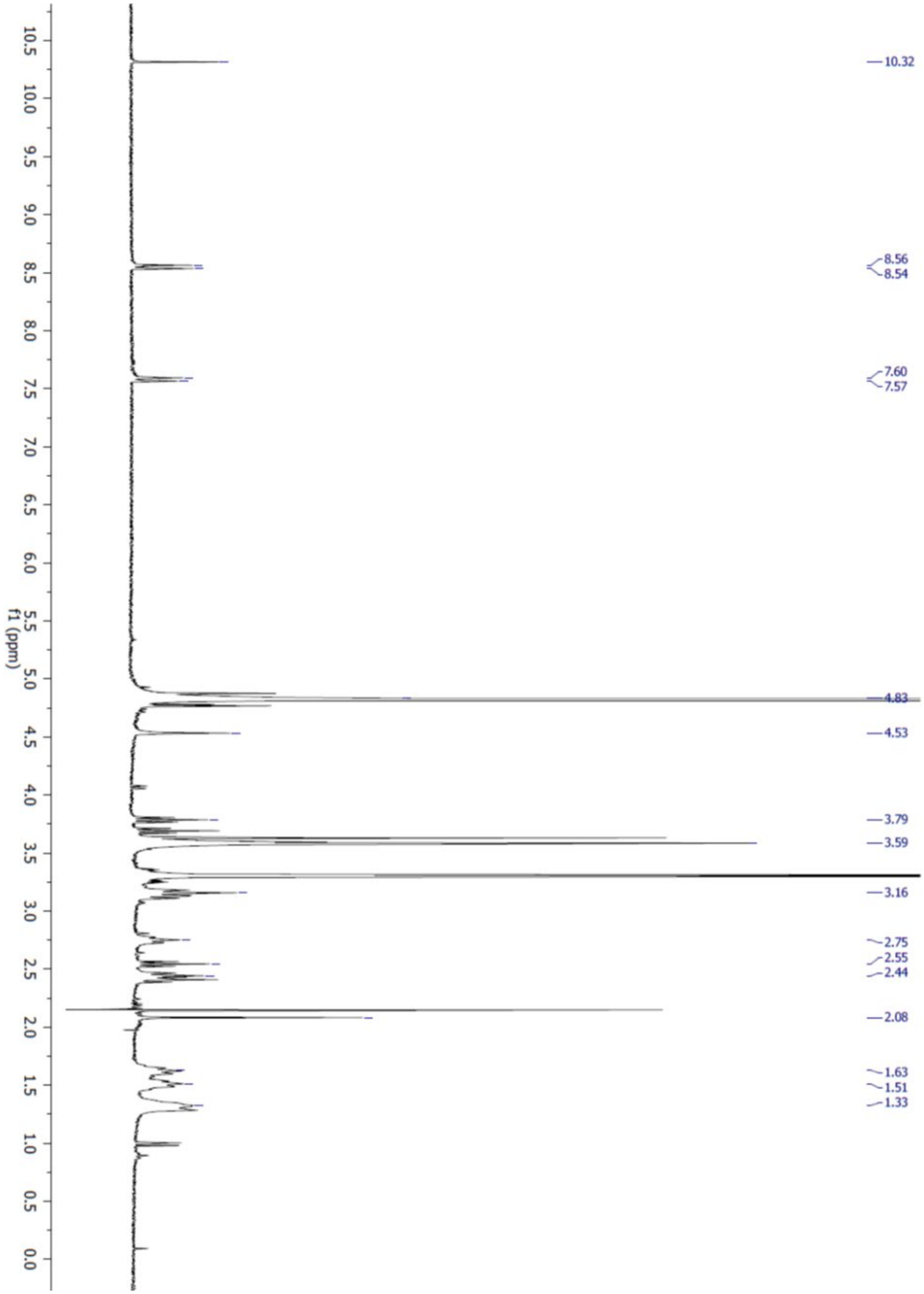

Figure S3. ^{1}H-NMR of DFO-PEG$_5$-Tz (**3**).

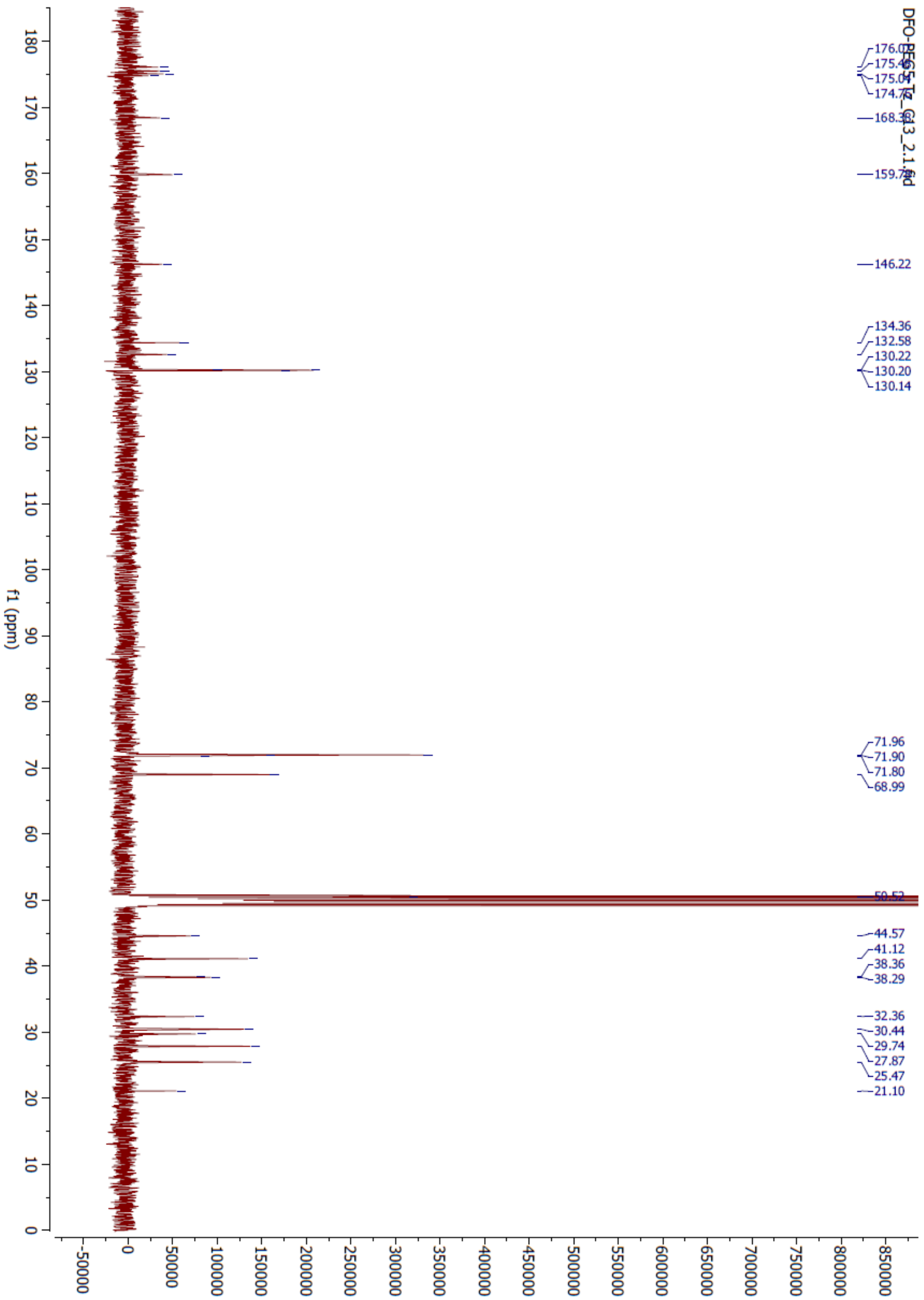

Figure S4. ^{13}C-NMR of DFO-PEG$_5$-Tz (**3**).

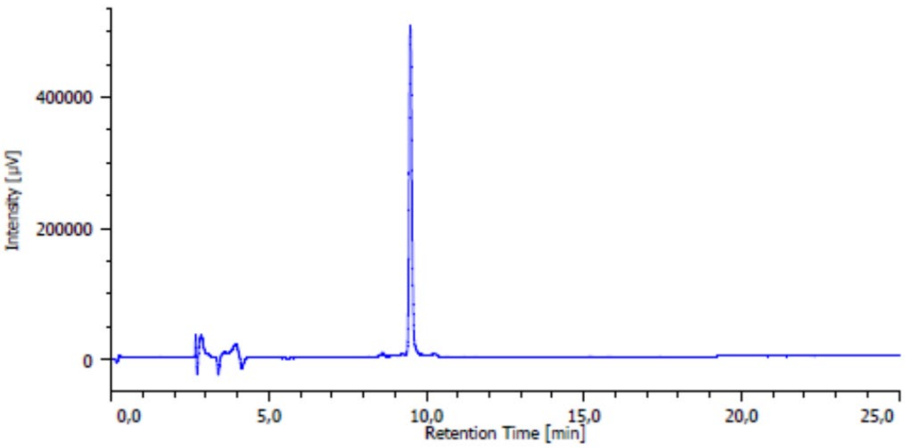

Figure S5. UV-HPLC chromatogram of [^{89}Zr]Zr-**3** in the formulation buffer (10% EtOH in saline+0.1 % Tween + 20 mM gentisic acid, pH 5.2).

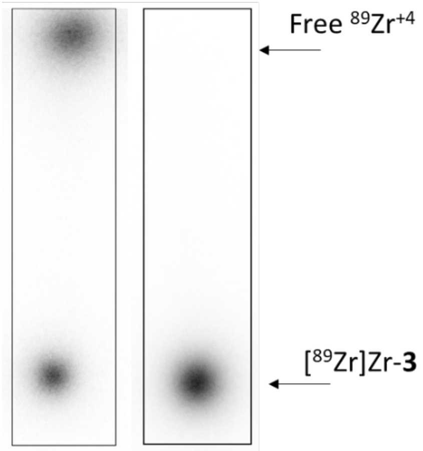

Figure S6. iTLC using 50 mM EDTA as an eluent from [^{89}Zr]Zr-**3** radiolabeling mixture before C18 SepPak purification (left) and after the purification from the purified [^{89}Zr]Zr-**3** (right).

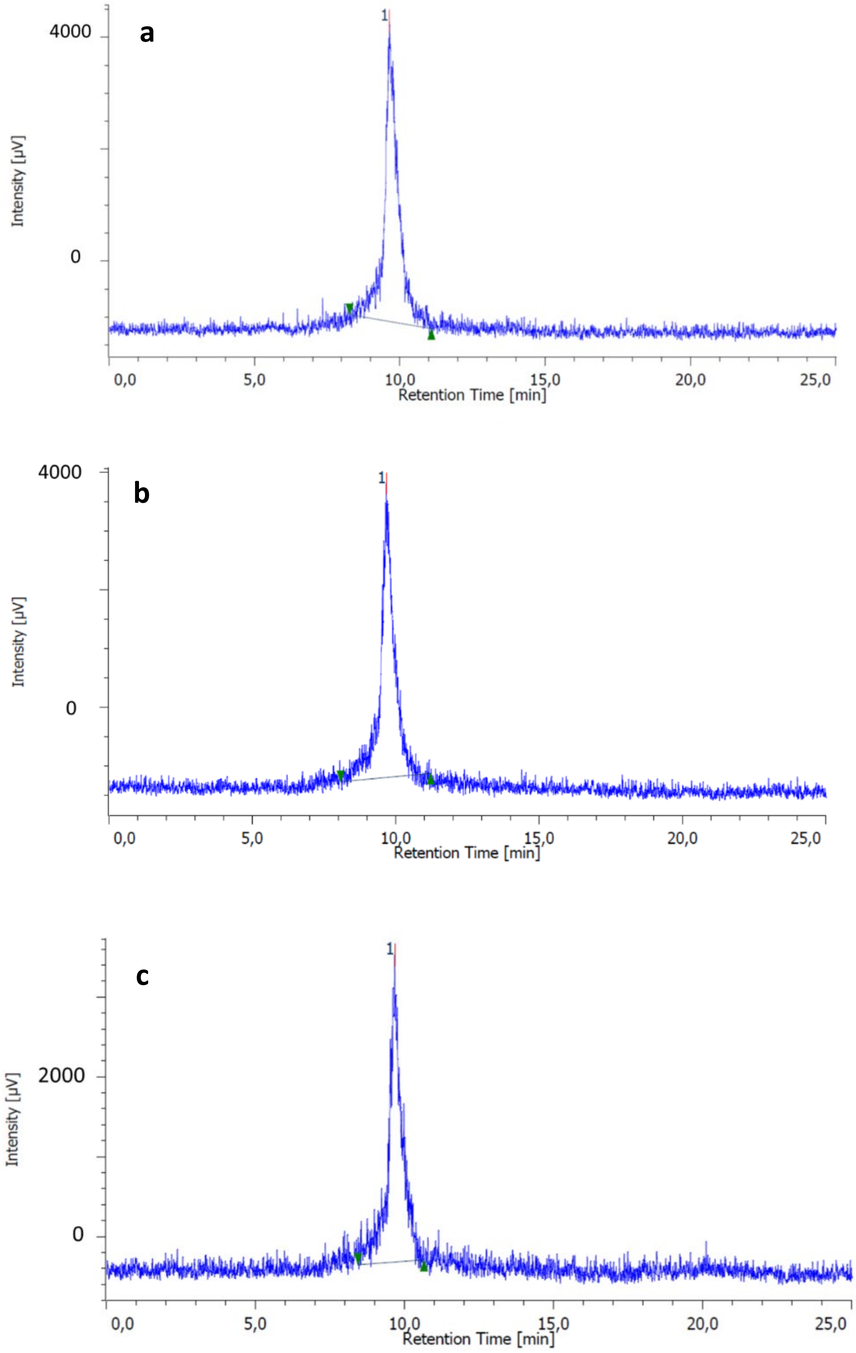

Figure S7. Radio-HPLC analysis of radiochemical stability of [^{89}Zr]Zr-**3** in the formulation buffer (10% EtOH in saline + 0.1 % Tween + 20 mM gentisic acid, pH 5.2) at a) 4 h, b) 24 h and c) 48 h time points.

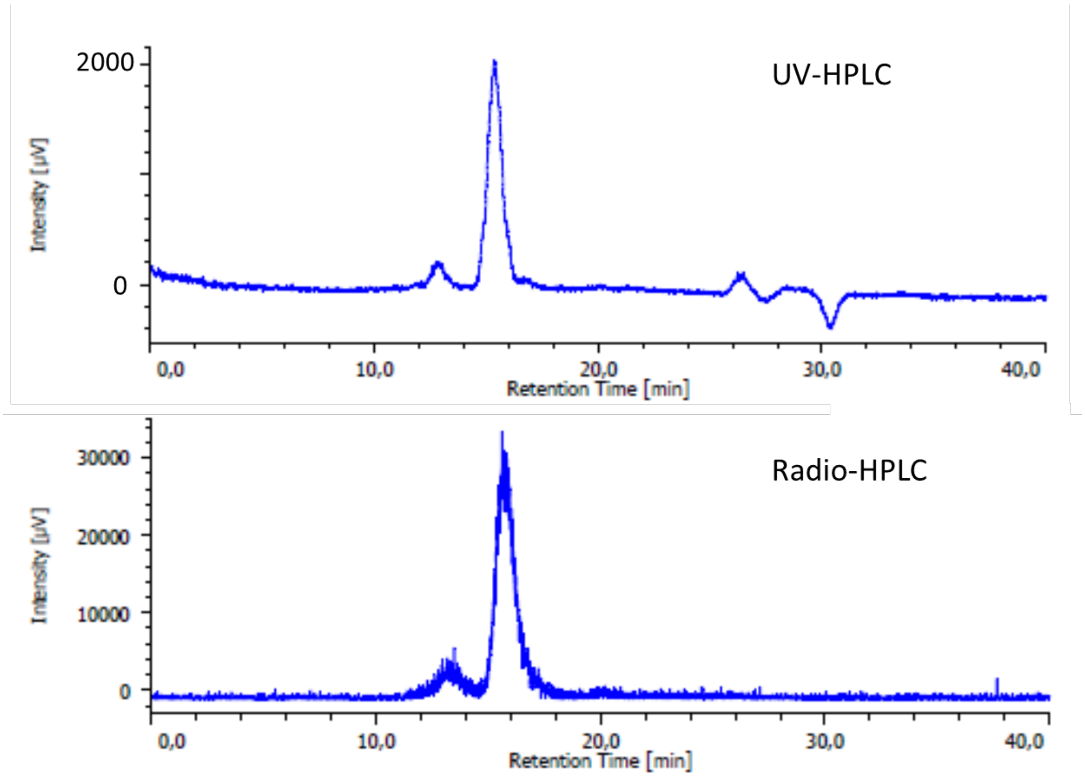

Figure S8. UV-HPLC chromatogram of TCO-U36 and radio-HPLC chromatogram of [^{89}Zr]Zr-**3**-TCO-U36 in PBS buffer. JASCO HPLC system with a Superdex® 200 Increase 10/300 GL (300 × 10 mm, 8.6 µm) size exclusion column (GE Healthcare Life Sciences) using 0.05 M phosphate buffer / 0.15 M NaCl / 0.01 NaN_3 (pH 6.7) as an eluent.

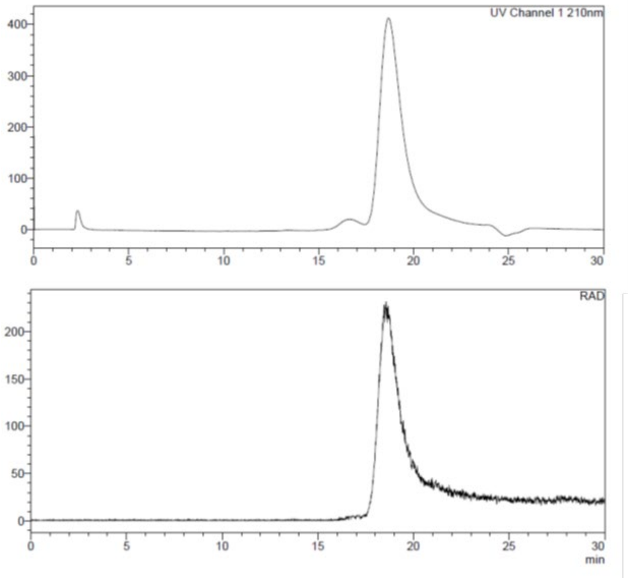

Figure S9. Radiochemical purity of [^{125}I]U36. UV-HPLC chromatogram and radio-HPLC chromatogram of [^{125}I]U36 (Phenomenex, Bio-Sep-SEC-s3060 (300 × 7.80 mm) with 0.05 M phosphate buffer / 0.15 M NaCl, pH 6.7).

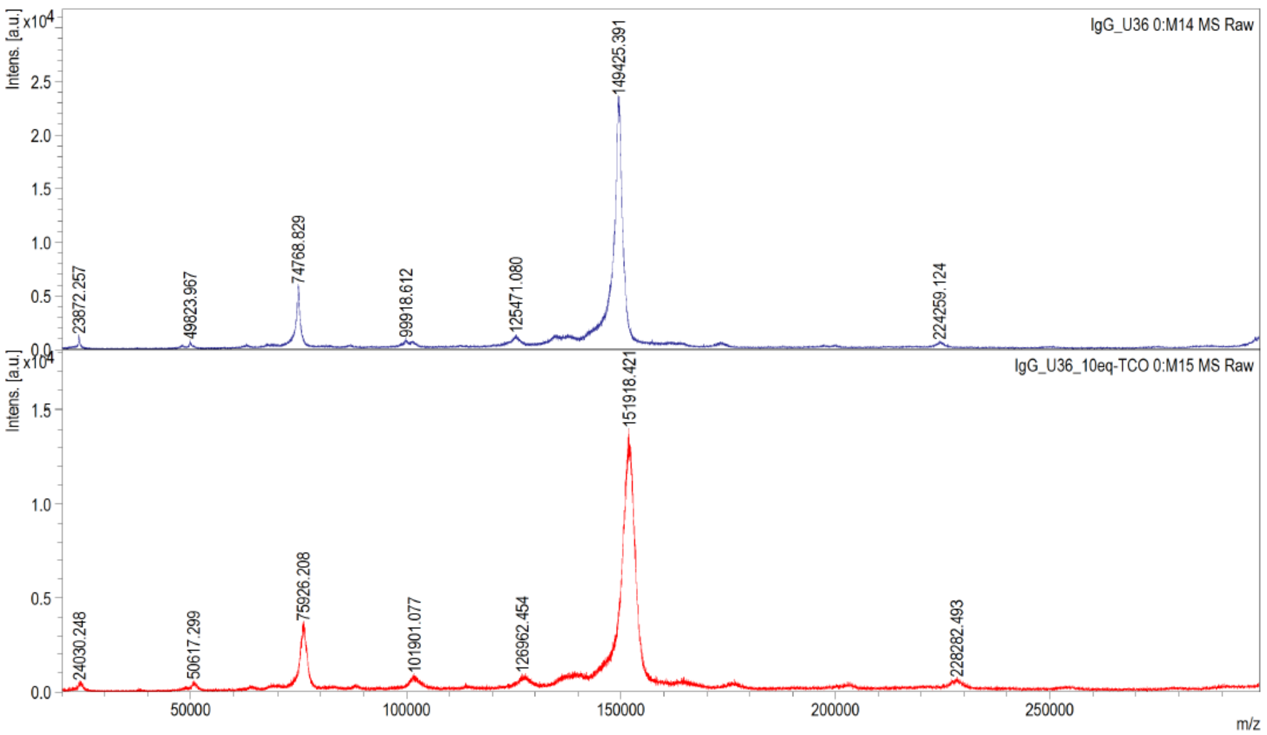

Figure S10. Mass spectra from MALDI analysis of U36 (upper) and U36 conjugated with 10 eq. of TCO (lower). Mass difference shows that the conjugation ratio is 6.2 TCO-to-U36.
